# Supplementary material for: Gli1+ progenitors mediate bone anabolic function of teriparatide via Hh and Igf signaling
Source: Cell Rep. Author manuscript; Available in PMC 2021 Sep 10. (PMC8432334; doi:10.1016/j.celrep.2021.109542)
Supplement: 1 [file NIHMS1734077-supplement-1.pdf]

Cell Reports, Volume *36*

Supplemental information

Gli1$^+$ progenitors mediate bone anabolic function of teriparatide via Hh and Igf signaling

Yu Shi, Xueyang Liao, James Y. Long, Lutian Yao, Jianquan Chen, Bei Yin, Feng Lou, Guangxu He, Ling Ye, Ling Qin, and Fanxin Long

Supplemental Information

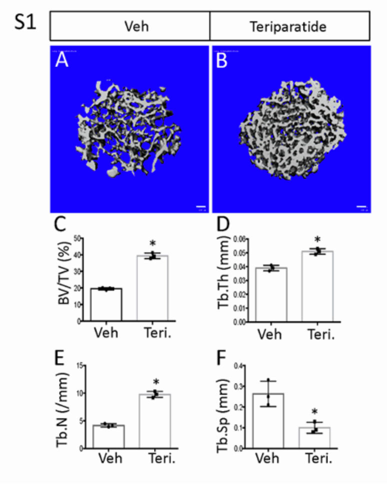

Figure S1. Related to Fig. 1. Teriparatide increases trabecular bone mass in growing mice.
One-month-old mice with the genotype of Gli1-CreER^{T2}; Ai9; Col1GFP were injected daily with teriparatide for 21 days after TAM induction.
(A, B) 3-D reconstruction of trabecular bone of the distal femur by microCT. Scale bars, 100 µm.
(C-F) Trabecular bone parameters of the distal femur by microCT. BV/TV, bone volume over total volume; Tb.Th, trabecular thickness; Tb.N, trabecular number; Tb.Sp, trabecular separation. *: $p<0.05$, n=3, student's t-test. Error bars: SD.

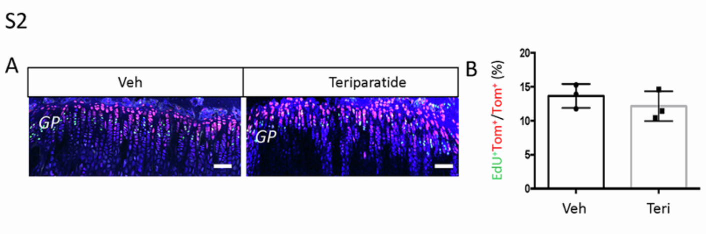

Figure S2. Related to Fig. 1. Teriparatide does not affect chondrocyte proliferation.
(A) Representative confocal images of the growth plate in proximal tibia of Gli1CreER^{T2};Ai9 mice injected with teriparatide or veh for 3 days after TM. Scale bar, 100 µm.
(B) Quantification of EdU labeling among tdTomato^{+} cells. N=3. Error bar: std.

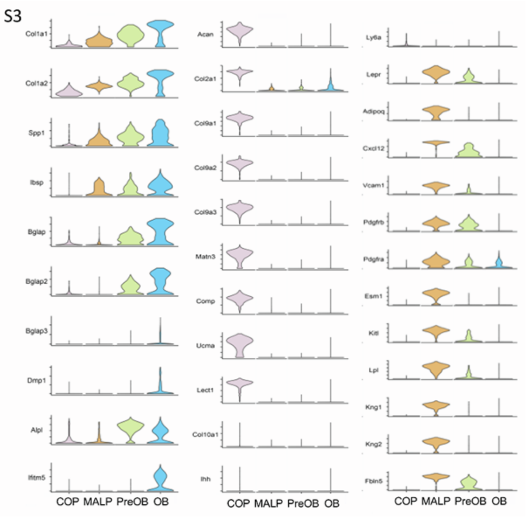

Figure S3. Related to Fig. 2. Violin plots of selected gene expression in various t-SNE clusters.

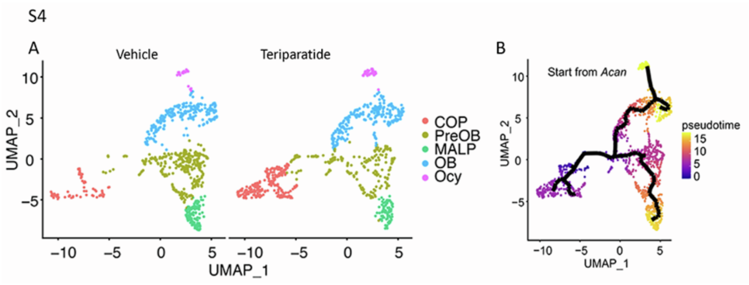

Figure S4. Related to Fig. 2. Clustering and trajectory analysis of scRNA-seq data.
(A) UMAP clustering showing expansion of COP population by teriparatide. (B) Trajectory analysis of integrated data based on with Monocle 3.

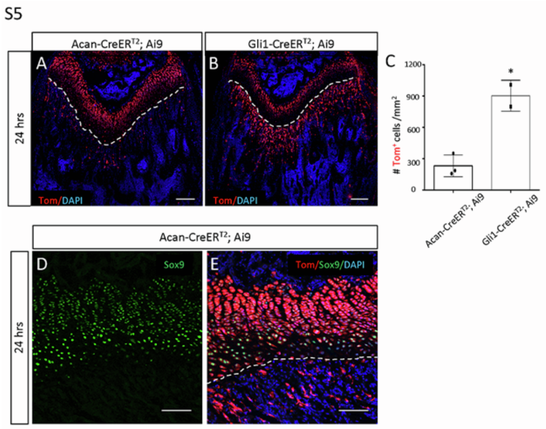

Figure S5. Related to Fig. 3. Acan-CreER^{T2} targets COP in primary spongiosa.
(A, B) Representative images of longitudinal sections through the distal end of femur from mice with indicated genotype. The mice were dosed with TAM once daily for three days starting at 28 days of age and harvested 24 hrs after last dosing. Dotted line denotes the boundary between growth plate and primary spongiosa. Scale bar: 200 µm.
(C) Quantification of tdTomato+ cells within the primary spongiosa region (200 µm below the growth plate and across the width from periosteum on one side to the other). *: $p<0.05$, student's t test, N= 2 or 3 as indicated. Error bars: SD.
(D, E) Immunofluorescence staining of Sox9 on longitudinal sections of distal femur from mice dosed in the same way as above. Dotted line denotes boundary between growth plate and primary spongiosa. Scale bar: 100 µm.

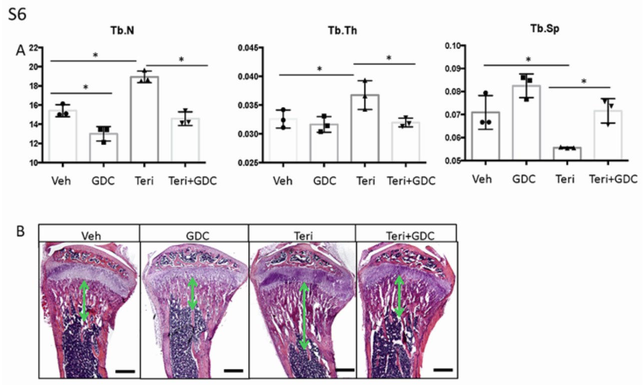

Figure S6. Related to Fig. 6. Hh inhibitor abolishes bone anabolic effect of teriparatide.
(A) Trabecular bone parameters of proximal tibia by µCT in wild type mice with indicated treatments for 10 days.
*: $p<0.05$, n=3, two-way ANOVA. Error bars: SD.

(B) H&E staining of longitudinal sections through the proximal tibia. Double-headed arrows denote trabecular bone region. Scale bar: 500 µm.

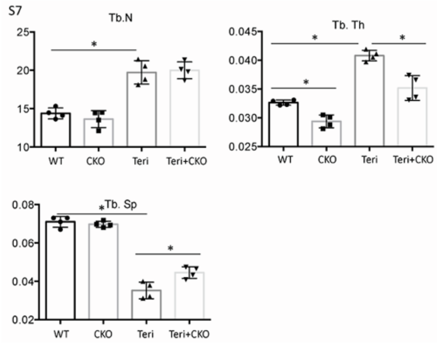

Figure S7. Related to Fig. 7. Igf signaling in MMPs mediates bone anabolic effect of teriparatide. Trabecular bone parameters in proximal tibia by µCT. Tb.Th, trabecular thickness; Tb.N, trabecular number; Tb. Sp, trabecular separation. *: $p<0.05$, n=4, two-way ANOVA. Error bars: SD.

Table S1. Osteoblast genes upregulated by teriparatide (adj. p value <0.05), related to figure 2

OB

Gene_name	Teriparatide	Ctrl	log2FoldChange	p value adj.
Mepe	9.0777407	1.5984315	2.40864179	3.27E-06
Dmp1	17.5897159	6.64350728	1.384023316	0.00072508
Spp1	318.871648	132.344947	1.267840155	0.00023216
Sost	0.18145602	4.16E-17	1.232158166	2.03E-06
Ibsp	35.1029097	17.2279159	1.023080885	7.48E-05
Phex	2.97786455	1.53991044	0.903718112	0.00580263
Igf1r	0.51172502	0.26877033	0.712284935	0.00061673
Runx3	0.24985181	0.11985354	0.685275545	0.01811181
Runx2	1.63327506	1.10661507	0.517875749	0.0049993

COP

Gene_name	Teriparatide	Ctrl	log2FoldChange	p value adj.
Spp1	79.6257212	5.6656089	3.765964542	3.57E-05
Bglap	7.12849537	1.5878098	2.064941224	0.00122549
Bglap2	3.61905627	0.9253039	1.805996461	0.01239873
Pth1r	21.857693	11.7368716	0.889462793	0.00122549
Mgp	1535.63859	830.105372	0.887362707	0.0015243
Runx3	8.44393483	5.02217675	0.727641422	0.00028417
Igf1r	3.55561895	2.44633204	0.501523514	0.00877632

MALP

Gene_name	Teriparatide	Ctrl	log2FoldChange	p value adj.
Igf1r	1.41764315	0.58445898	1.12093741	1.83E-06
Runx3	0.4429948	0.20982488	0.755811899	0.03278384
Runx2	3.38584968	2.06855003	0.66985307	0.00018058
Col1a2	32.5810338	21.5872848	0.591683594	0.0001039
Runx1	9.41439148	7.29164917	0.356351682	0.04347791

PreOB

Gene_name	Teriparatide	Ctrl	log2FoldChange	p value adj.
Ibsp	35.1973347	2.87881181	3.553566065	5.14E-18
Spp1	158.306014	15.2091273	3.370175069	6.12E-22
Mgp	7.29253118	1.42200914	2.265824032	2.74E-10
Mmp13	28.2094678	5.82654048	2.245066959	2.72E-09
Enpp1	0.60317565	0.02809828	2.222976672	1.06E-08
Dmp1	0.52975239	1.39E-17	2.191031879	1.14E-05
Postn	8.55677376	2.27527289	1.859864679	4.84E-06
Col1a2	116.207557	41.9731649	1.466451914	4.43E-08
Sparc	61.3568624	24.71528	1.307552181	7.71E-07
Col1a1	136.120292	57.6667035	1.23744936	0.00079425
Bgn	13.8732873	6.28057894	1.125676081	1.56E-09
Runx1	2.69878894	1.4830065	0.815731883	0.00199852

Table S2. Gene Ontology Enrichment by Teriparatide in COP, Related to Figure 2

Annotation Cluster 1	Enrichment Score: 40.72	Count	P_Value	Fold Change	Benjamini
UP_KEYWORDS	Ribonucleoprotein	66	1.70E-49	1.20E+01	4.90E-47
KEGG_PATHWAY	Ribosome	54	1.10E-47	1.40E+01	2.10E-45
UP_KEYWORDS	Ribosomal protein	55	6.30E-47	1.50E+01	9.00E-45
GOTERM_MF_DIRECT	Structural constituent of ribosome	56	3.10E-39	1.00E+01	1.60E-36
GOTERM_CC_DIRECT	Ribosome	49	6.50E-39	1.30E+01	2.10E-36
GOTERM_BP_DIRECT	Translation	53	2.00E-26	6.30E+00	3.60E-23
**Annotation Cluster 2**	**Enrichment Score: 18.24**	**Count**	**P_Value**	**Fold Change**	**Benjamini**
KEGG_PATHWAY	Oxidative phosphorylation	37	3.20E-26	9.80E+00	3.00E-24
KEGG_PATHWAY	Parkinson's disease	34	9.90E-22	8.40E+00	6.20E-20
UP_KEYWORDS	Mitochondrion inner membrane	33	1.10E-17	7.00E+00	5.40E-16
KEGG_PATHWAY	Non-alcoholic fatty liver disease	30	6.60E-17	7.10E+00	3.10E-15
KEGG_PATHWAY	Huntington's diease	32	7.90E-16	6.00E+00	3.00E-14
KEGG_PATHWAY	Alzheimer's disease	30	2.10E-15	6.30E+00	6.40E-14
**Annotation Cluster 3**	**Enrichment Score: 16.89**	**Count**	**P_Value**	**Fold Change**	**Benjamini**
UP_KEYWORDS	Respiratory chain	21	6.50E-20	1.80E+01	4.70E-18
GOTERM_CC_DIRECT	Respiratory chain	19	5.70E-17	1.60E+01	3.10E-15
UP_KEYWORDS	Electron transport	22	5.70E-16	1.10E+01	2.30E-14

Table S3. Gene Ontology Enrichment by Teriparatide in MALP, Related to Figure 2

Annotation Cluster 1	Enrichment Score: 45.39	Count	P_Value	Fold Change	Benjamini
KEGG_PATHWAY	Ribosome	68	4.40E-58	1.20E+01	9.60E-56
UP_KEYWORDS	Ribosomal protein	69	3.40E-55	1.20E+01	1.00E-52
GOTERM_CC_DIRECT	Ribosome	65	2.10E-49	1.10E+01	9.20E-47
UP_KEYWORDS	Ribonucleoprotein	74	2.40E-47	8.80E+00	3.70E-45
GOTERM_MF_DIRECT	structural constituent of ribosome	71	2.40E-45	8.60E+00	1.60E-42
GOTERM_CC_DIRECT	intracellular ribonucleoprotein complex	65	7.20E-34	6.60E+00	1.50E-31
GOTERM_BP_DIRECT	translation	72	1.40E-33	5.70E+00	3.60E-30
**Annotation Cluster 2**	**Enrichment Score: 13.37**	**Count**	**P_Value**	**Fold Change**	**Benjamini**
KEGG_PATHWAY	Oxidative phosphorylation	36	6.20E-20	6.80E+00	6.80E-18
KEGG_PATHWAY	Parkinson's disease	32	3.40E-15	5.60E+00	2.50E-13
KEGG_PATHWAY	Alzheimer's disease	32	4.90E-13	4.70E+00	2.70E-11
KEGG_PATHWAY	NAFLD	28	2.60E-11	4.70E+00	1.20E-09
KEGG_PATHWAY	Huntington's disease	31	5.40E-11	4.10E+00	2.00E-09
**Annotation Cluster 3**	**Enrichment Score: 12.65**	**Count**	**P_Value**	**Fold Change**	**Benjamini**
UP_KEYWORDS	Respitatory chain	20	2.00E-15	1.20E+01	1.20E-13
GOTERM_CC_DIRECT	respiratory chain	19	6.40E-14	1.10E+01	3.10E-12
UP_KEYWORDS	Electron transport	20	8.30E-11	6.80E+00	3.60E-09

Table S4. Gene Ontology Enrichment by Teriparatide in PreOB, Related to Figure 2

Annotation Cluster 1	Enrichment Score: 24.87	Count	P_Value	Fold Change	Benjamini
UP_KEYWORDS	Ribonucleoprotein	55	3.70E-28	6.50E+00	6.50E-26
UP_KEYWORDS	Ribosomal protein	46	4.00E-28	8.30E+00	6.50E-26
KEGG_PATHWAY	Ribosome	45	1.30E-26	7.30E+00	3.10E-24
GOTERM_CC_DIRECT	ribosome	42	2.00E-23	7.20E+00	3.40E-21
GOTERM_MF_DIRECT	structural constituent of ribosome	47	1.10E-21	5.70E+00	8.00E-19
Annotation Cluster 2	Enrichment Score: 8.41	Count	P_Value	Fold Change	Benjamini
INTERPRO	Collagen triple helix repeat	18	5.00E-11	8.10E+00	5.80E-08
UP_KEYWORDS	Collagen	18	1.20E-10	7.80E+00	3.80E-09
GOTERM_CC_DIRECT	collagen trimer	18	5.30E-10	7.00E+00	1.90E-08
KEGG_PATHWAY	Protein digestion and absorption	14	7.40E-05	3.80E+00	1.40E-03
Annotation Cluster 3	Enrichment Score: 7.82	Count	P_Value	Fold Change	Benjamini
KEGG_PATHWAY	Oxidative phosphorylation	27	9.70E-11	4.60E+00	1.10E-08
KEGG_PATHWAY	Alzheimer's disease	28	4.80E-09	3.70E+00	2.80E-07
KEGG_PATHWAY	Parkinson's disease	25	1.20E-08	4.00E+00	5.60E-07
KEGG_PATHWAY	Huntington's disease	24	9.20E-06	2.90E+00	2.40E-04

Table S5. Gene Ontology Enrichment by Teriparatide in OB, Related to Figure 2

Annotation Cluster 1	Enrichment Score: 30.53	Count	P_Value	Fold Change	Benjamini
UP_KEYWORDS	Ribonucleoprotein	57	4.70E-36	8.80E+00	1.40E-33
UP_KEYWORDS	Ribosomal protein	48	2.10E-35	1.10E+01	3.10E-33
KEGG_PATHWAY	Ribosome	47	3.50E-34	1.00E+01	6.90E-32
GOTERM_MF_DIRECT	Structural constituent of ribosome	51	6.60E-31	8.20E+00	3.90E-28
GOTERM_CC_DIRECT	Ribosome	44	2.70E-30	1.00E+01	1.00E-27
GOTERM_BP_DIRECT	Translation	49	1.00E-20	5.20E+00	2.20E-17
Annotation Cluster 2	Enrichment Score: 8.61	Count	P_Value	Fold Change	Benjamini
UP_KEYWORDS	Respiratory chain	14	4.10E-10	1.10E+01	2.10E-08
GOTERM_CC_DIRECT	Respiratory chain	14	6.60E-10	1.00E+01	2.50E-08
UP_KEYWORDS	Electron transport	15	5.30E-08	6.70E+00	1.80E-06
Annotation Cluster 3	Enrichment Score: 2.61	Count	P_Value	Fold Change	Benjamini
INTERPRO	Ribonucleoprotein LSM domain	5	9.00E-04	1.10E+01	2.10E-01
SMART	Sm	5	1.10E-03	1.10E+01	2.30E-01
INTERPRO	Like-Sm (LSM) domain	5	1.60E-03	9.70E+00	2.10E-01
GOTERM_CC_DIRECT	U12-type spliceosomal complex	4	2.50E-02	6.30E+00	2.50E-01
