# Supplementary material for: Gli1+ progenitors mediate bone anabolic function of teriparatide via Hh and Igf signaling
Source: Cell Rep. Author manuscript; Available in PMC 2021 Sep 10. (PMC8432334; doi:10.1016/j.celrep.2021.109542)
Supplement: 2 [file NIHMS1734077-supplement-2.pdf]

Cell Reports

Article

# Gli1$^{+}$ progenitors mediate bone anabolic function of teriparatide via Hh and Igf signaling

## Graphical abstract

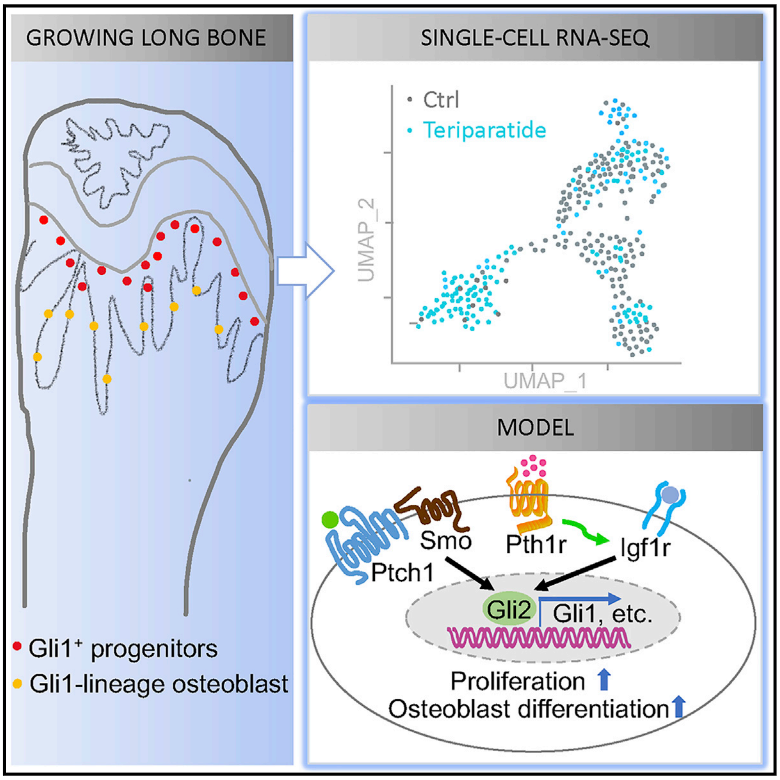


## Highlights

- Teriparatide promotes proliferation and osteoblast differentiation of Gli1$^{+}$ progenitors
- scRNA-seq uncovers chondrocyte-like osteoprogenitors (COPs) among Gli1$^{+}$ progenitors
- COPs are target cells of teriparatide
- Hh and Igf signaling in Gli1$^{+}$ progenitors mediates the anabolic effect of teriparatide


## Authors

Yu Shi, Xueyang Liao, James Y. Long, ..., Ling Ye, Ling Qin, Fanxin Long

## Correspondence

longf1@chop.edu



## In brief

Gli1$^{+}$ metaphyseal mesenchymal progenitors (MMPs) are a main source for osteoblasts in postnatal mice. Through single-cell RNA sequencing, Shi et al. find a chondrocyte-like osteoprogenitor (COP) among MMPs. COP is a target of teriparatide, the mainstay of bone anabolic therapy, and requires Hh and Igf signaling for bone formation.


Shi et al., 2021, Cell Reports 36, 109542
August 17, 2021 © 2021 The Author(s).
https://doi.org/10.1016/j.celrep.2021.109542

CellPress

Cell Reports

CellPress
OPEN ACCESS

Article

# Gli1$^+$ progenitors mediate bone anabolic function of teriparatide via Hh and Igf signaling

Yu Shi,[1,2] Xueyang Liao,[1,2] James Y. Long,[3] Lutian Yao,[2] Jianquan Chen,[4] Bei Yin,[1] Feng Lou,[5] Guangxu He,[6] Ling Ye,[1] Ling Qin,[7] and Fanxin Long[2,7,8,*]
[1]State Key Laboratory of Oral Diseases & National Clinical Research Center for Oral Diseases, West China Hospital of Stomatology, Sichuan University, Chengdu, China
[2]Translational Research Program of Pediatric Orthopedics, Department of Surgery, Children's Hospital of Philadelphia, Philadelphia, PA, USA
[3]Courant Institute of Mathematical Sciences, New York University, New York, NY, USA
[4]Orthopedic Institute, Medical College, Soochow University, Suzhou, China
[5]West China Hospital of Stomatology, Sichuan University, Chengdu, China
[6]Department of Orthopedics, The Second Xiangya Hospital, Central South University, Changsha, China
[7]Department of Orthopedic Surgery, University of Pennsylvania, Philadelphia, PA, USA
[8]Lead contact
*Correspondence: longf1@chop.edu
https://doi.org/10.1016/j.celrep.2021.109542

## SUMMARY

Teriparatide is the most widely prescribed bone anabolic drug in the world, but its cellular targets remain incompletely defined. The Gli1$^+$ metaphyseal mesenchymal progenitors (MMPs) are a main source for osteoblasts in postnatal growing mice, but their potential response to teriparatide is unknown. Here, by lineage tracing, we show that teriparatide stimulates both proliferation and osteoblast differentiation of MMPs. Single-cell RNA sequencing reveals heterogeneity among MMPs, including an unexpected chondrocyte-like osteoprogenitor (COP). COP expresses the highest level of Hedgehog (Hh) target genes and the insulin-like growth factor 1 receptor (Igf1r) among all cell clusters. COP also expresses Pth1r and further upregulates Igf1r upon teriparatide treatment. Inhibition of Hh signaling or deletion of Igf1r from MMPs diminishes the proliferative and osteogenic effects of teriparatide. The study therefore identifies COP as a teriparatide target wherein Hh and insulin-like growth factor (Igf) signaling are critical for the osteoanabolic response in growing mice.

## INTRODUCTION

Osteoblasts (OBs), the chief bone-making cells, are replenished throughout life in mammals, but their origin in postnatal life remains to be fully elucidated (Long, 2011). Lineage-tracing experiments have identified the Gli1-positive metaphyseal mesenchymal progenitors (MMPs) as a major source for trabecular osteoblasts in the long bones of mice up to four months of age (Shi et al., 2017). Besides osteoblasts, MMPs give rise to the leptin receptor-positive (LepR$^+$) bone marrow stromal cells, which in turn have been shown to produce osteoblasts in adult mice progressively with age (Mizoguchi et al., 2014; Zhou et al., 2014a). Thus, MMPs are not only immediate osteoblast precursors in the growing mice but may also provide long-term osteoprogenitors persisting in the bone marrow later in life.

Teriparatide, a recombinant peptide corresponding to the N-terminal fragment (amino acids 1–34) of human parathyroid hormone (PTH), has been the mainstay of bone anabolic therapy for nearly two decades (Cipriani et al., 2012). Once-daily injections of teriparatide stimulate bone formation in humans and mice alike (Esen et al., 2015). Extensive studies in mice have indicated that PTH or teriparatide promotes bone formation through direct regulation of osteoblast-lineage cells at multiple levels, including promotion of osteoblast activity, stimulation of osteoblast differentiation, attenuation of osteoblast apoptosis, and activation of quiescent bone-lining cells (Jilka, 2007; Kim et al., 2012). More recently, teriparatide was shown to increase both number and osteoblast differentiation of a Sox9-positive osteoprogenitor population located within the primary spongiosa of growing mice (Balani et al., 2017). Because previous immunostaining did not detect Sox9 protein in MMPs, it is not known whether MMPs would similarly respond to the anabolic regimen of teriparatide (Shi et al., 2017) .

Indian Hedgehog (Ihh), one of the three Hedgehog (Hh) proteins in mammals, is a critical regulator of osteoblast differentiation during endochondral bone development (St-Jacques et al., 1999). Like all Hh proteins, Ihh signals via the seven-pass transmembrane protein Smoothened (Smo) to modulate gene expression by activating or de-repressing the Gli family (Gli1–Gli3) of transcription factors (Ingham and McMahon, 2001). Gli1 is both an effector and a transcriptional target of Hh signaling, thus serving as a useful marker for Hh-responding cells. Genetic studies of Smo in the mouse have demonstrated a direct requirement for Hh signaling in osteoblast differentiation in


This is an open access article under the CC BY license (http://creativecommons.org/licenses/by/4.0/).

the long bones; the osteoblastogenic effect is mediated by both de-repression of Gli3 and activation of Gli2 (Joeng and Long, 2009; Long et al., 2001). Furthermore, Hh signaling via Gli2 was shown to activate insulin-like growth factor (Igf) signaling in a positive feedback mechanism to induce osteoblast differentiation (Shi et al., 2015). However, a potential role for Igf signaling in the Hh-responding MMPs is yet to be determined.

Here we show that once-daily injection of teriparatide promotes both proliferation and osteoblast differentiation of MMPs to enhance bone formation in growing mice. The effects of teriparatide on MMPs require both Ihh and Igf signaling. Single-cell RNA sequencing (scRNA-seq) has uncovered heterogeneity among MMPs, including a chondrocyte-like osteoprogenitor (COP) responsive to Hh and teriparatide signaling during bone formation.

## RESULTS

### Teriparatide stimulates bone formation from Gli1$^+$ progenitors

To investigate the potential effect of teriparatide on the Gli1$^+$ MMPs *in vivo*, we genetically marked the cells and their progenies with the red fluorescent protein tdTomato before subjecting the mice to once-daily treatment of teriparatide. Mice with a genotype of Gli1-CreER$^{T2}$;tdTomato;ColI-GFP were administered tamoxifen (TAM) once daily for three consecutive days starting at one month of age and then injected with teriparatide daily for 21 days before harvest. Micro-computed tomography (μCT) imaging and quantification of the cancellous bone in the distal femur confirmed that teriparatide caused a notable increase in bone mass (BV/TV), trabecular thickness (Tb.th), and trabecular bone number (Tb.N), coupled with decreased trabecular spacing (Tb.Sp), compared to the vehicle injection (Figure S1). When the bone sections were examined for tdTomato marking the Gli1-lineage cells, we found that teriparatide greatly increased the number of tdTomato+ cells within the metaphyseal trabecular bone region (Figures 1A–1C). Teriparatide also extended the red domain within the metaphyseal periosteum but had no obvious effect on the labeling of the growth plate or the articular cartilage (Figures 1A and 1B). More importantly, teriparatide markedly increased the number of the GFP$^+$ tdTomato$^+$ cells (yellow) within the trabecular bone region, indicating a greater number of mature osteoblasts derived from MMPs (Figures 1A1, 1B1, and 1D). Thus, teriparatide not only expands the total population of MMPs and progenies but also increases the number of osteoblast descendants.

### Teriparatide stimulates proliferation and osteoblast differentiation of Gli1$^+$ progenitors

To examine the acute effect of teriparatide on MMPs, we injected the Gli1-CreER$^{T2}$;tdTomato mice with teriparatide once daily for three days after marking the cells with TAM at one month of age. The mice were harvested at one day after the last teriparatide dose, and EdU (5-ethynyl-2'-deoxyuridine) was injected shortly before sacrifice to assess cell proliferation *in vivo*. Quantification of the EdU$^+$ cells on sections through the bone metaphysis revealed that teriparatide increased the proliferation index among tdTomato$^+$ cells (Figures 1E–1G). The proliferative effect was specific to MMPs or their progenies, because teriparatide did not alter EdU labeling of the Gli1-lineage cells within the growth plate (Figure S2). In addition, immunostaining for Osx showed that a higher percentage of Gli1-lineage cells expressed the osteoblast marker upon teriparatide treatment (Figures 1H–1J). However, TUNEL staining detected no change in the rate of apoptosis among the Gli1-lineage cells (9.3% ± 3.2% for vehicle versus 9.5% ± 2.3% for teriparatide, n = 3), even though teriparatide reduced overall apoptosis within the metaphyseal marrow compartment (45.1 ± 3.8/section for vehicle versus 27.3 ± 6.1/section for teriparatide, $p < 0.05$, n = 3 mice, one section per mouse). The data therefore indicate that teriparatide stimulates both proliferation and osteogenic differentiation of MMPs or their progenies.

To examine the effect of teriparatide more directly on MMPs, we treated the mice first with teriparatide once daily for three days before marking the MMPs with TAM. To exclude the possibility that teriparatide might simply activate the Gli1-CreER$^{T2}$ allele in mature osteoblasts, we performed the experiment in Gli1-CreER$^{T2}$;tdTomato;ColI-GFP mice wherein the mature osteoblasts expressed GFP and therefore would turn yellow if they activated Gli1-CreER$^{T2}$. Teriparatide expanded the tdTomato$^+$ population in the primary spongiosa but did not increase the small percentage (~10%) of yellow cells normally seen among MMPs (Figures 1K–1N). Thus, the increase in Gli1$^+$ cells likely resulted from direct expansion of MMPs instead of Gli1-CreER$^{T2}$ activation in mature osteoblasts. To assess directly the effect on the proliferation status, we performed EdU labeling in Gli1-CreER$^{T2}$;Ai9 mice that received teriparatide for three days before TAM for one day. The EdU labeling index among tdTomato+ cells was increased by teriparatide (Figures 1O–1Q). Thus, teriparatide expands the pool size of MMPs through stimulation of cell proliferation.

### scRNA-seq identifies chondrocyte-like osteoprogenitors as a teriparatide target

To gain further insights about MMPs, we performed single-cell RNA sequencing with or without teriparatide treatment. Specifically, we treated one-month-old Gli1-CreER$^{T2}$;tdTomato mice with three daily doses of TAM and then three daily injections of teriparatide or vehicle before sacrifice on the following day (6 days from the first TAM injection). After removal of the growth plate, the metaphyseal trabecular bone was enzymatically digested; tdTomato$^+$CD45$^-$Ter119$^-$CD31$^-$ cells were selected from the dissociated cells by flow cytometry. The single cells were then subjected to RNA sequencing with 10x Genomics. After excluding the residual contamination of hematopoietic cells expressing Ptprc (encoding CD45), integrated analysis of the vehicle and teriparatide datasets identified four major clusters by tSNE (t-distributed stochastic neighbor embedding), including osteoblasts (OBs) marked by Sp7, Col1a1, Bglap2, and Dmp1 (Figures 2A and 2B; Figure S3). A second cluster expressed early osteoblast genes such as Runx2, Sp7, and Postn, but not Dmp1, and therefore was designated preosteoblast (preOB) (Figures 2B and 2C). The third cluster was enriched for chondrocyte markers, including Sox9, Sox5, Sox6, Col2a1, and Acan (Figures 2B and 2C). Because the growth plate was discarded before single-cell isolation and the hypertrophic chondrocytes that were closest to the

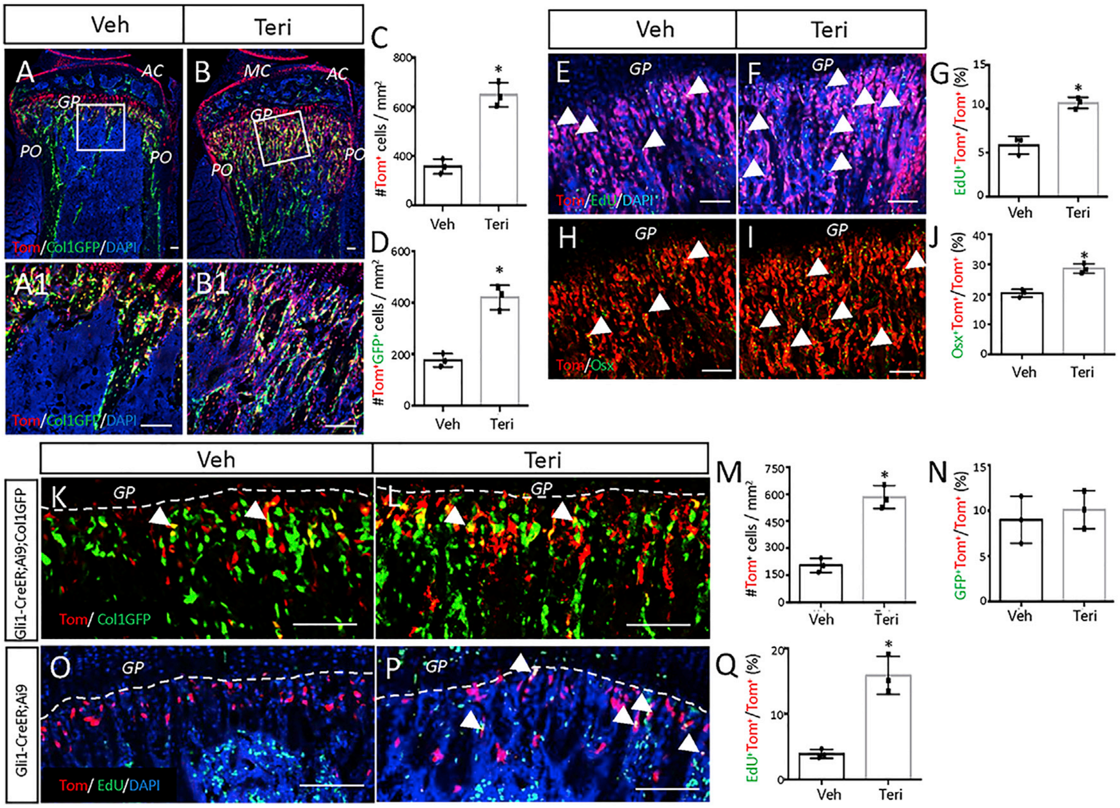

**Figure 1. Teriparatide promotes proliferation and osteoblast differentiation of MMPs**

(A–D) Mice with the genotype of Gli1-CreER$^{T2}$;Ai9;ColI-GFP were injected daily with teriparatide (Teri) or vehicle (Veh) for 21 days after TAM induction. (A and B) Representative confocal images of the proximal tibia. MC, meniscus; AC, articular cartilage; GP, growth plate; PO, periosteum. Boxed areas are shown below at a higher magnification (A1 and B1). tdTomato, GFP, and DAPI were visualized by direct fluorescence. Same below. DAPI stains nuclei of all cells. (C and D) Number of tdTomato+ (C) or tdTomato$^+$GFP$^+$ cells (D) in the primary spongiosa region extending 300 μm from the growth plate and spanning the width of the bone flanked by the periosteum. The same region of interest applies to quantifications below. ImageJ was used for all quantifications.

(E–J) Mice with the genotype of Gli1-CreER$^{T2}$;Ai9 were injected with teriparatide or vehicle for 3 days after TAM. (E, F, H, and I) Representative confocal images of primary spongiosa in the proximal tibia showing colocalization of tdTomato with EdU (E and F) or Osx (H and I). EdU was detected by click reaction, and Osx was detected by immunostaining. Arrowheads denote double-positive cells. (G and J) Quantification of EdU-positive cells (G) or Osx-positive cells (J) among tdTomato$^+$ cells in the primary spongiosa region as described in (C) and (D).

(K–N) Mice with the genotype of Gli1-CreER$^{T2}$;Ai9;ColI-GFP were injected with teriparatide or vehicle for three days before TAM for three days. (K and L) Representative confocal images of primary spongiosa in the proximal tibia. (M and N) Quantification of total tdTomato$^+$ (M) or the percentage of tdTomato$^+$ cells expressing GFP (N) in the primary spongiosa region as described in (C) and (D).

(O–Q) Mice with the genotype of Gli1-CreER$^{T2}$;Ai9 were injected with teriparatide or vehicle for three days before TAM for one day. (O and P) Representative confocal images of primary spongiosa in the proximal tibia. (Q) Quantification of the EdU-labeling percentage among tdTomato$^+$ cells in the primary spongiosa region as described in (C) and (D). Dashed lines demarcate the boundary of the growth plate. Arrows denote double-positive cells.

Scale bars: 100 μm. All quantifications were performed within the primary spongiosa region. Error bars: SD. *$p < 0.05$, n = 3 mice, 2 sections for each mouse, Student's t test.

trabecular bone region did not express tdTomato, we considered those chondrocyte-like cells unlikely to be conventional growth-plate chondrocytes. Furthermore, because most of the cluster also expressed Runx2 (81.2% of the cells) and Sp7 (60.7%), which are indicative of osteoblastogenic potentials, we termed the cluster COP (Figure 2C). The final cluster expressed Lepr, Cxcl12, Vcam1, Pdgfrb, and Adipoq, among other markers, and appeared to be identical to the adiponectin-expressing bone marrow stromal cells recently designated as marrow adipogenic lineage progenitors (MALPs) (Mukohira et al., 2019; Zhong et al., 2020) (Figure 2B; Figure S3). Thus, scRNA-seq has uncovered heterogeneous cell clusters among MMPs.

Further analysis of the clusters revealed unique features of COP. They were enriched for the prototypical Hh target genes Gli1, Ptch1, and Hhip, indicating that they were most responsive to Hh signaling among the clusters at the time of isolation (Figure 2D). Although all clusters expressed the teriparatide receptor Pth1r, COP was further enriched for the Igf signaling components including Igf1r, Irs1, and Irs2, whereas MALP expressed the most Igf1 (Figure 2E). Interestingly, teriparatide expanded the COP cluster, apparently at the expense of MALP and preOB (Figure 2F) (see Discussion). However, analysis of the differential gene expression showed that teriparatide increased the expression of multiple osteoblast marker genes in all four clusters, indicating

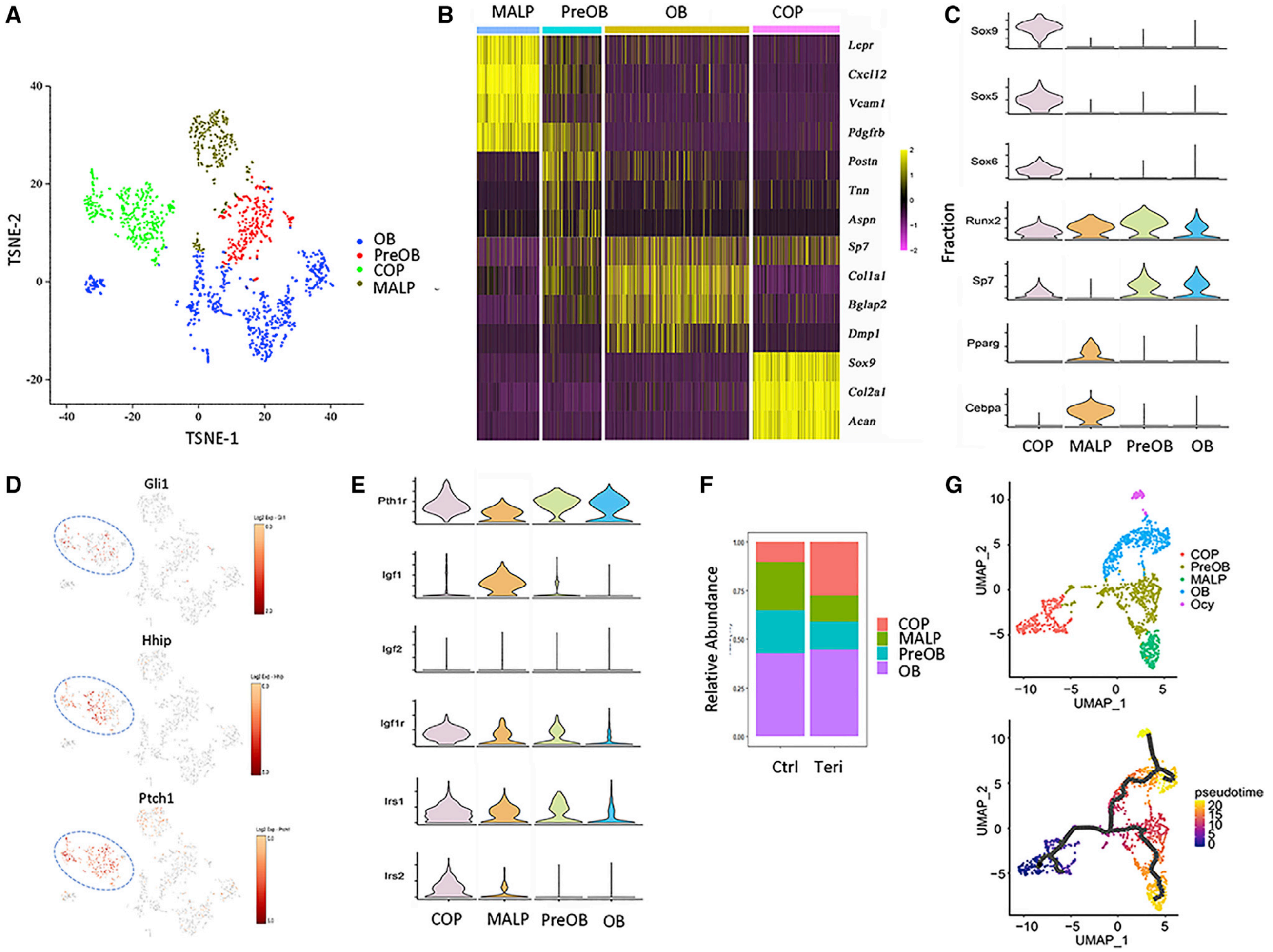


**Figure 2. scRNA-seq reveals heterogeneity of MMPs**

(A) Visualization of clusters from integrated analysis of vehicle and teriparatide datasets.

(B) Heatmap of representative marker genes for each cluster.

(C and E) Violin plots of representative genes for each cluster.

(D) Enriched expression of Hh target genes in the COP cluster.

(F) Relative abundance of each cluster in MMPs of vehicle- versus teriparatide-treated mice. OB, osteoblast; preOB, preosteoblast; COP, chondrocyte-like osteoprogenitor; MALP, marrow adipocyte lineage progenitor.

(G) Clustering by UMAP (upper) and subsequent pseudotime analysis with Monocle 3 (lower).

a broad osteogenic effect of teriparatide (Table S1). In particular, Igf1r was upregulated by teriparatide in the COP, OB, and MALP clusters, supporting a potential role of the Igf pathway in mediating teriparatide signaling. Furthermore, Gene Ontology and pathway analysis with DAVID (the database for annotation, visualization and integrated discovery) identified ribosomal proteins and oxidative phosphorylation as the top categories induced by teriparatide across all clusters (Tables S2–S5). Thus, teriparatide exerts a broad osteogenic and anabolic effect on all MMPs and their progenies, including the expansion of COP.

To explore the potential lineage relationship among clusters, we next performed trajectory analysis with Monocle 3. Clustering of the integrated data by UMAP (uniform manifold appropriation and projection) confirmed the same four clusters as found by tSNE, except that it distinguished a subgroup of the osteoblast cluster as Ocy marked by the expression of Dmp1 and Mepe (Figure 2G, upper). Separate visualization of the datasets confirms that teriparatide expanded the COP cluster (Figure S4A). Trajectory prediction based on Sox9 or Acan expression revealed temporal progression from COP to preOB and further to either MALP or OB/Ocy (Figure 2G, lower; Figure S4B). The computational analysis therefore indicates that COP could potentially function as osteoblast and MALP progenitors.

To test directly the computational prediction of COP as osteoblast progenitors, we used the Acan-CreER$^{T2}$ mouse that expresses CreER$^{T2}$ from the endogenous aggrecan gene locus and is therefore expected to mark the COP cells upon TAM administration. We first dosed the Acan-CreER$^{T2}$;Ai9 mice with TAM at four weeks of age before harvesting them 24 h later. Compared

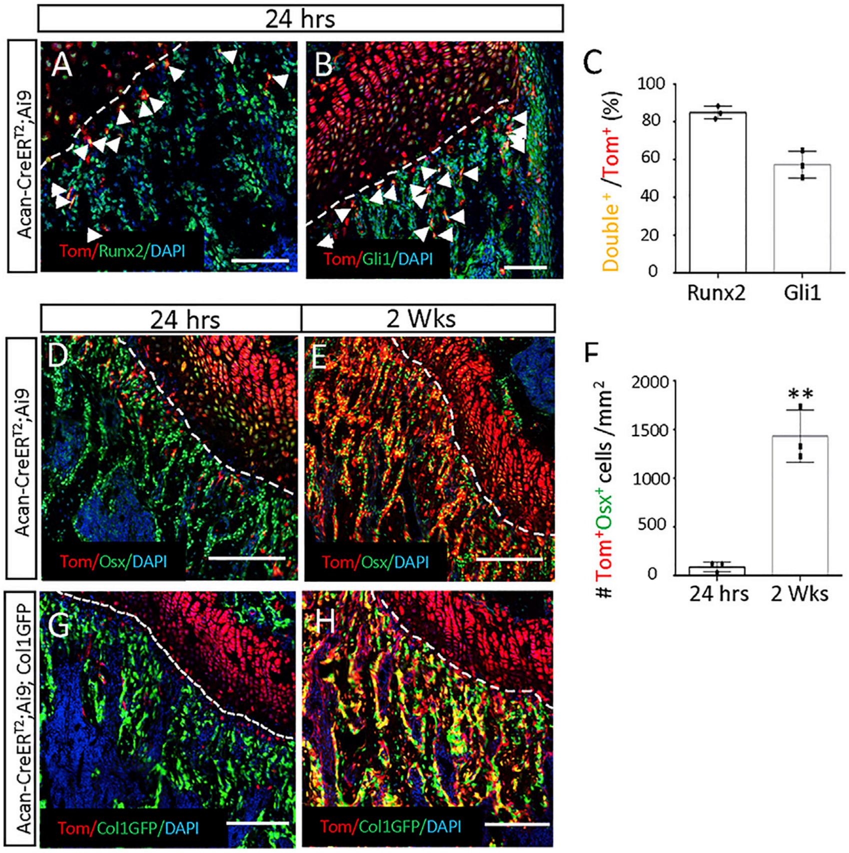


**Figure 3. COP gives rise to osteoblasts in the mouse**

Acan-CreER$^{T2}$;Ai9 mice (A–F) or Acan-CreER$^{T2}$;Ai9;Coll-GFP mice (G and H) were treated with TAM for three days at 28 days of age and harvested after 24 h or 2 weeks.

(A and B) Representative confocal images showing direct fluorescence of tdTomato and immunostaining of Runx2 (A) or Gli1 (B) in the distal femur of Acan-CreER$^{T2}$;Ai9 mice harvested at 24 h after TAM. DAPI stains all nuclei. Nonnuclear staining from the Gli1 antibody is likely nonspecific. Arrowheads point to double-positive cells in the primary spongiosa. Dotted line denotes the boundary between the growth plate and the primary spongiosa. Same below.

(C) Quantification of double-positive cells among tdTomato+ cells in the primary spongiosa region represented in (A) and (B). Region of interest (ROI) extends 200 μm below the growth plate and spans the width of bone. Image Pro Plus was used for quantification.

(D and E) Representative confocal images showing direct fluorescence of tdTomato and immunostaining of Osx in the distal femur of Acan-CreER$^{T2}$;Ai9 mice harvested at 24 h (D) or 2 weeks (E) after TAM.

(F) Quantification of double-positive cells in the primary spongiosa region as represented in (D) and (E). Same ROI as in (C). Error bars: SD. **$p < 0.01$, $n = 3$, Student's t test.

(G and H) Representative confocal images showing direct fluorescence of tdTomato and GFP in the distal femur of Acan-CreER$^{T2}$;Ai9;Coll-GFP mice harvested at 24 h (G) or 2 weeks (H) after TAM.

Scale bars: 100 μm (A and B), 200 μm (D, E, G, and H).

with Gli1-CreER$^{T2}$, Acan-CreER$^{T2}$ marked significantly fewer cells within the primary spongiosa (Figures S5A–S5C). Immunostaining detected Runx2 or Gli1 protein in most Acan-CreER$^{T2}$-targeted cells in the primary spongiosa, thus confirming the utility of Acan-CreER$^{T2}$ in targeting COP cells (Figures 3A–3C). However, Sox9 immunostaining failed to detect a clear signal in the primary spongiosa, indicating that the COP cells may contain little Sox9 protein despite mRNA expression; the result confirms our previous finding (Figures S5D and S5E) (Shi et al., 2017). We next tracked the fate of the COP cells for two weeks in the Acan-CreER$^{T2}$;Ai9 mice after TAM dosing at 4 weeks of age. Immunostaining for Osx showed that compared with 24 h, the tdTomato+Osx+ cells greatly expanded in the primary spongiosa and trabecular bone region (Figures 3D–3F). To clarify whether COP targeted by Acan-CreER$^{T2}$ became mature osteoblasts with time, we repeated the lineage tracing experiment in mice with the genotype of Acan-CreER$^{T2}$;Ai9;Coll-GFP. At 24 h, the tdTomato$^+$ COP cells were distinct from the GFP+ mature osteoblasts (Figure 3G). However, after 2 weeks, many double-positive cells were detected within the trabecular bone region, providing direct evidence for the differentiation of COP to osteoblasts (Figure 3H). The lineage tracing experiments therefore confirm the prediction from trajectory analysis that COP is an osteoblast progenitor.

We next examined the effect of teriparatide on COP. Mice with the genotype of Acan-CreER$^{T2}$;Ai9 were dosed with TAM at four weeks of age and then injected with teriparatide or vehicle once daily for three days before harvest. The mice were injected with EdU shortly before sacrifice to allow for cell proliferation assays. Immunostaining for Osx showed that teriparatide increased both the total number of tdTomato$^+$ Osx$^+$ cells and the percentage of double positives among tdTomato+ cells within the primary spongiosa (Figures 4A–4D). Moreover, EdU labeling detected an increase in proliferation among the tdTomato+ cells by teriparatide (Figures 4E–4G). To determine whether teriparatide enhances the production of mature osteoblasts from COP, we treated the Acan-CreER$^{T2}$;Ai9;Coll-GFP mice with teriparatide or vehicle for seven days after they were dosed with TAM at four weeks of age. Teriparatide markedly increased the number of tdTomato+GFP+ osteoblasts within the trabecular bone region (Figures 4H–4J). Thus, genetic experiments demonstrate that teriparatide stimulates COP to proliferate and produce more osteoblasts *in vivo*.

## Hh signal is required for teriparatide stimulation of MMPs

Because MMPs are characterized by Hh signaling and stimulated by teriparatide, we next examined whether the two signals interact in the progenitors. Ihh expressed by growth-plate chondrocytes is the predominant Hh ligand in the growing skeleton, so we decided to test whether reduced Ihh signaling might change the effect of teriparatide. Although the full knockout of

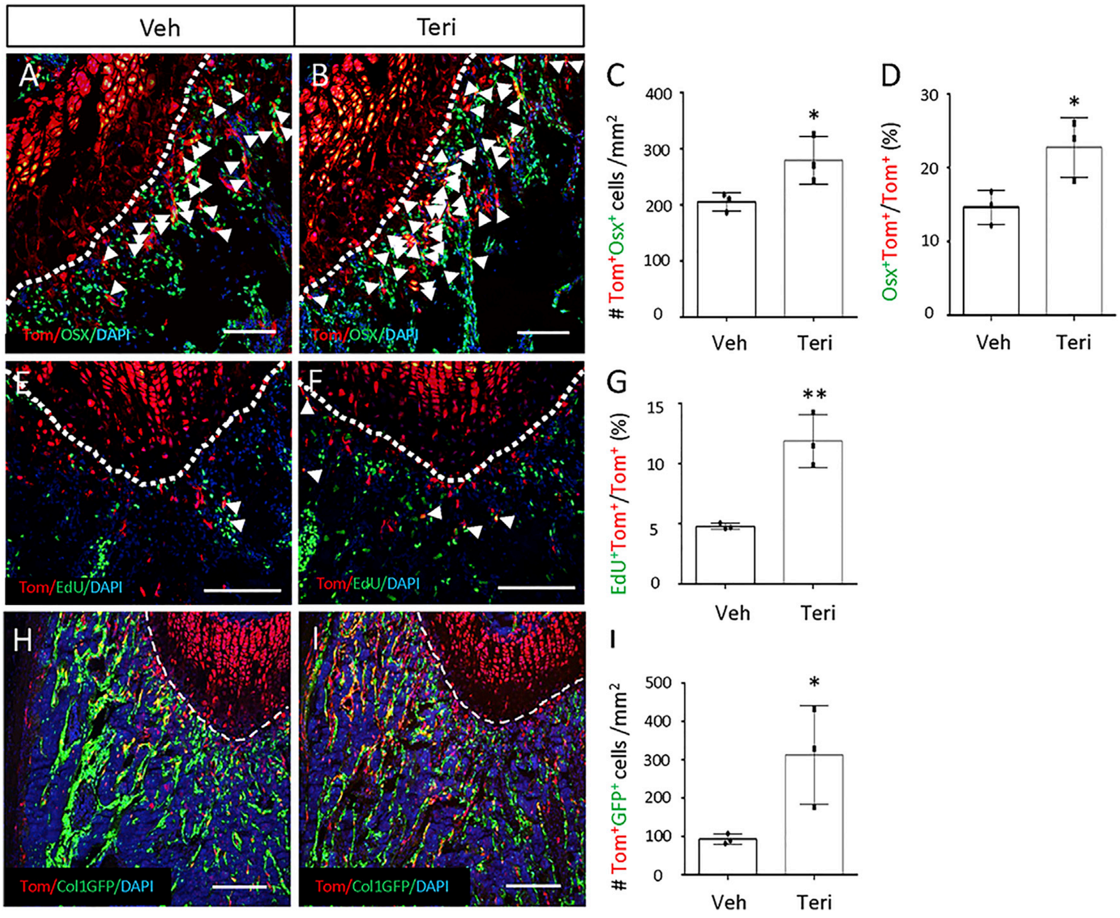


**Figure 4. Teriparatide stimulates COP proliferation**

(A–G) Acan-CreER$^{T2}$;Ai9 mice were treated with TAM and then injected with teriparatide or vehicle for 3 days. (A and B) Confocal images showing tdTomato direct fluorescence and Osx immunostaining on sections of the distal femur. Arrowheads denote double-positive cells in the primary spongiosa. Dotted lines denote the boundary of the growth plate. Same below. (C and D) Quantification of double-positive cells in the primary spongiosa as represented in (A) and (B). The region for quantification extends 200 μm below the growth plate and spans the width of the bone flanked by the periosteum. Same below. ImageJ used for quantification. (E and F) Confocal images showing tdTomato$^+$EdU$^+$ cells (arrowheads) in the primary spongiosa of the distal femur. (G) EdU labeling index of tdTomato$^+$ cells in the primary spongiosa as represented in (E) and (F). Region of quantification is the same as in (C) and (D).

(H–J) Mice with the genotype of Acan-CreER$^{T2}$;Ai9;ColI-GFP were treated with TAM and then injected with teriparatide or vehicle for 7 days. (H and I) Confocal images showing tdTomato and GFP direct fluorescence in the primary spongiosa region of the distal femur. (J) Quantification of tdTomato$^+$GFP$^+$ cells in the primary spongiosa region as represented in (H) and (I). Region for quantification is the same as in (C) and (D).

Scale bars: 100 μm. Error bars: SD. *$p < 0.05$, **$p < 0.01$, n = 3, Student's t test.

Ihh leads to neonatal lethality, heterozygous mice live without obvious defects (St-Jacques et al., 1999). Therefore, we produced mice with the genotype of Gli1-CreER$^{T2}$;Ai9;Ihh$^{+/-}$ and the littermate control of Gli1-CreER$^{T2}$;Ai9 to compare their response to teriparatide followed by TAM administration at 1 month of age. When the animals were injected the vehicle solution, the number of tdTomato$^+$ cells within the metaphysis was similar between the genotypes (Figures 5A, 5A1, 5B, and 5B1). As expected, teriparatide greatly expanded the tdTomato$^+$ population in the control mice (Figures 5A, 5A1, 5C, and 5C1). However, no such increase was observed in the Gli1-CreER$^{T2}$;Ai9;Ihh$^{+/-}$ mice (Figures 5B, 5B1, 5D, and 5D1). These results indicate that teriparatide expands the pool of MMPs, likely by amplifying Hh signaling among mesenchymal cells within the primary spongiosa.

We next assessed the effect of Hh signaling on MMP proliferation and differentiation induced by teriparatide. GDC-0449, a potent inhibitor of Smo, was used to block Hh signaling with or without teriparatide coinjection for three days, following TAM administration in the Gli1-CreER$^{T2}$;Ai9 mice. EdU staining showed that GDC-0449 not only decreased the basal proliferation rate of MMPs but also eliminated the mitogenic effect of teriparatide (Figures 6A and 6B). Similarly, the inhibitor abolished the induction of Osx in MMPs by teriparatide (Figures 6C and 6D). Thus, Hh signaling is essential for teriparatide to stimulate the proliferation and osteoblast differentiation of MMPs.

To assess the impact of Hh inhibition on the bone anabolic effect of teriparatide, we treated wild-type mice with teriparatide, GDC-0449, or both for ten days. μCT analyses revealed that GDC-0449 modestly reduced the normal cancellous bone mass

CellPress
OPEN ACCESS

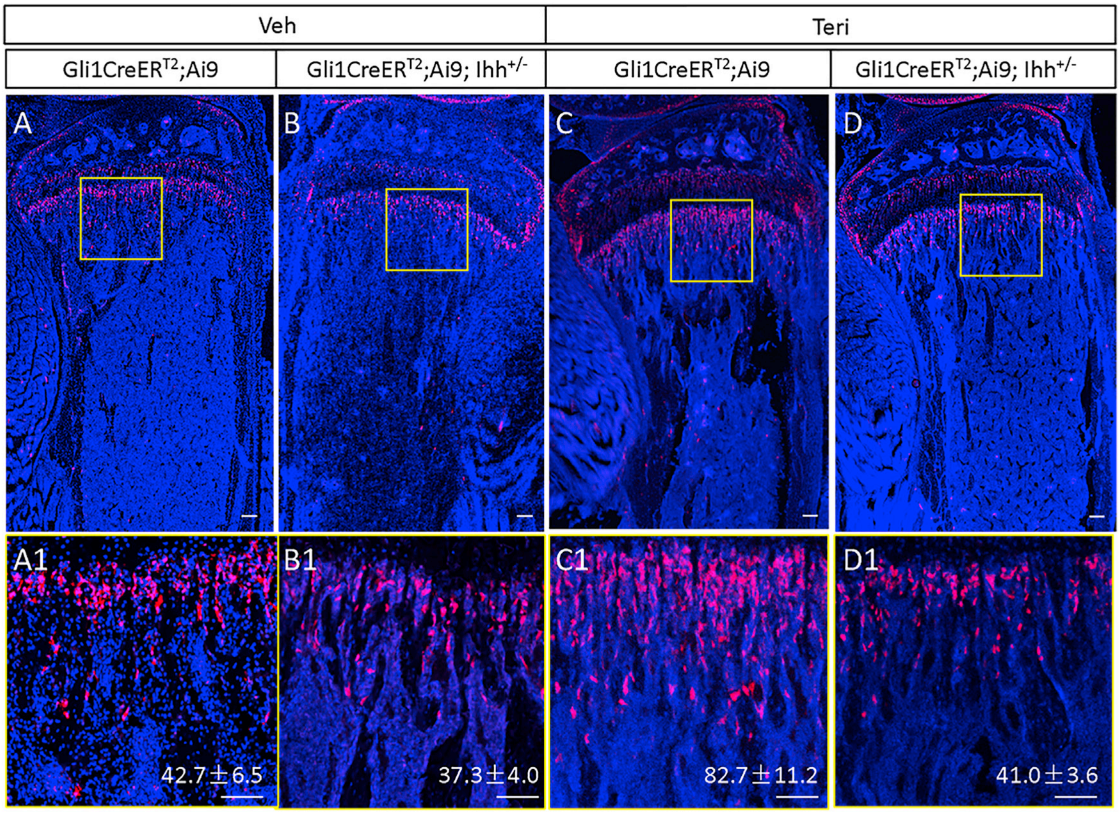


**Figure 5. Ihh is required for teriparatide stimulation of MMPs and progenies**

Mice of indicated genotypes were injected at one month of age with teriparatide or vehicle for three days and then TAM for three days.

(A–D) Confocal images of longitudinal sections through the proximal tibia.

(A1–D1) Boxed regions in (A)–(D) shown at higher magnification. Red, tdTomato$^+$ MMPs and progenies; blue, DAPI nuclear staining. Scale bar: 100 μm. tdTomato$^+$ cells were counted in a primary spongiosa area of 0.09 mm$^2$ (300 × 300 μm) immediately below the growth plate. Quantification indicates mean cell number ± SD, n = 3. One section per mouse was quantified.

but essentially eliminated the increase caused by teriparatide (Figures 6E and 6F; Figure S6A). Histology of the tibia confirmed that GDC-0449 abolished the expansion of the cancellous bone region by teriparatide (Figure S6B). Serum biochemistry showed that the inhibitor significantly suppressed the circulating PINP (procollagen type I N-terminal propeptide) level, an indicator of overall bone formation activity (Figure 6G). However, GDC-0449 did not seem to affect the bone resorption marker CTX-I (type I collagen cross-linked c-telopeptide), which was elevated by teriparatide as expected (Figure 6H). Because teriparatide is known to increase the activity of existing osteoblasts, we performed double-labeling experiments to assess whether GDC-0449 also affected osteoblast function. Quantification showed that the stimulation of mineral apposition rate (MAR) by teriparatide on the endosteal surface of the tibia was not affected by GDC-0449, indicating that the suppression of serum PINP levels by the inhibitor mostly resulted from decreased *de novo* osteoblast production (Figures 6I and 6J). Potential minor effects of GDC-0449 on bone resorption or osteoblast activity could still exist but were not detected here because of the small sample size. Nonetheless, the data support that Hh signaling is required for teriparatide to stimulate osteoblast differentiation from MMPs.

## Igf signaling is required for teriparatide to stimulate bone formation from MMPs

Because COP is enriched for the Igf signaling components and upregulates Igf1r expression in response to teriparatide, we next investigated whether Igf and teriparatide signaling interact in MMPs. To this end, we generated mice with the genotype of Gli1-CreER$^{T2}$;Ai9;Igf1r$^{c/c}$ (CKO) to allow simultaneous labeling of MMPs and deletion of Igf1r upon TAM administration. We first assessed the impact of Igf1r deletion on the acute effect of teriparatide. For this, the CKO and control littermates with the genotype of Gli1-CreER$^{T2}$;Ai9;Igf1r$^{c/+}$ (Ctrl) were treated with teriparatide or vehicle for three days following TAM dosing at one month of age. EdU labeling indicated that Igf1r deletion reduced the basal rate of MMP proliferation; it also blunted, but did not eliminate, induction by teriparatide (Figures 7A and 7B). Statistical analysis with two-way ANOVA did not detect a significant interaction between Igf1r and teriparatide (p value 0.8856), indicating that the two signals may stimulate proliferation independently. However, because we have not determined the deletion efficiency of Igf1r in MMPs, we cannot rule out that the apparent lack of interaction may result from incomplete deletion of Igf1r. Deletion of Igf1r also reduced the percentage of

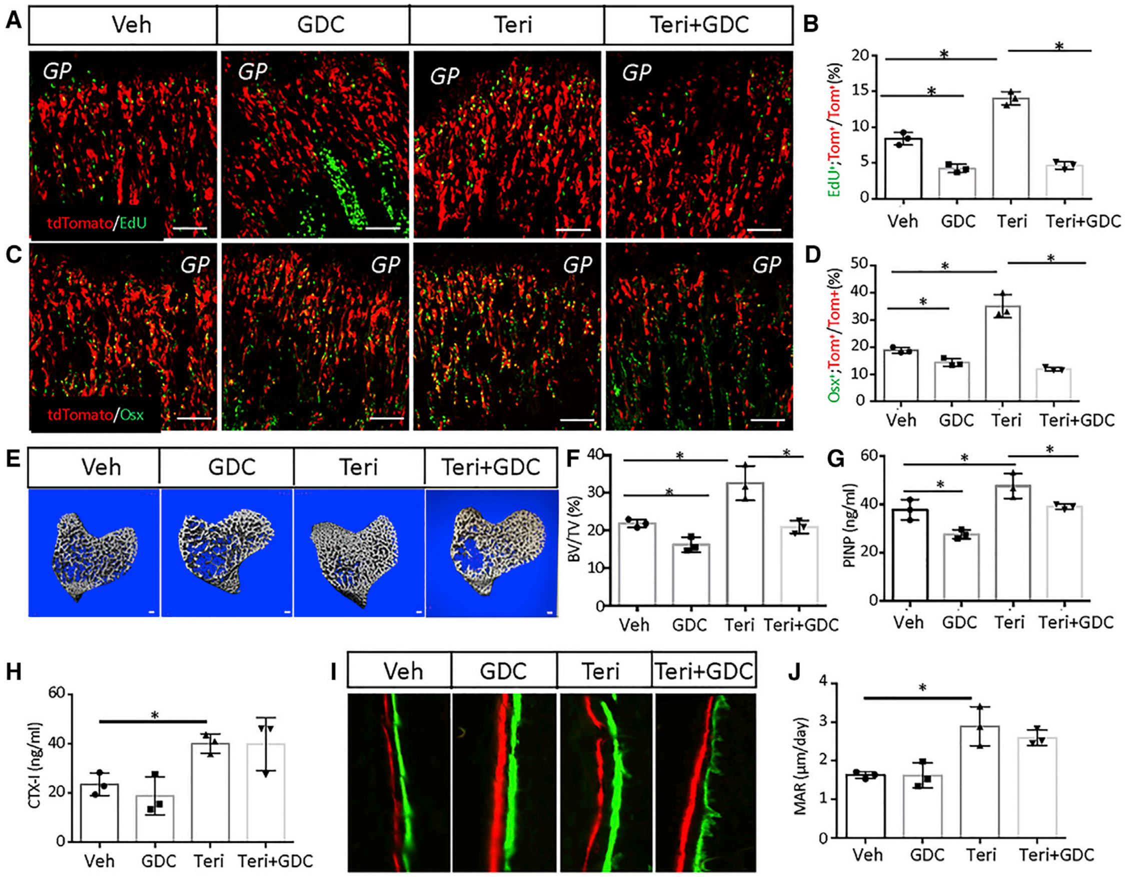


**Figure 6. Hh signal is required for proliferative and osteogenic effects of teriparatide**

(A and C) Representative confocal images of the chondro-osseous junction in the distal femur. Gli1-CreER$^{T2}$;Ai9 mice were first treated with TAM and then injected with GDC, teriparatide, or both for 3 days. Scale bar: 100 μm. PBS was used as vehicle control.

(B and D) Quantification of tdTomato$^{+}$EdU$^{+}$ (B) or tdTomato$^{+}$Osx$^{+}$ (D) over tdTomato$^{+}$ cells in areas represented by (A) or (C), respectively. Percentage (mean ± SD) was quantified within the primary spongiosa of the proximal tibia, extending 300 μm from the growth plate and across the width of the bone in two sections per mouse and three mice per group.

(E) μCT images of cancellous bone of the proximal tibia in wild-type mice with indicated treatments for 10 days. Scale bar: 100 μm.

(F) Quantification of the trabecular bone mass (BV/TV) by μCT.

(G and H) Serum levels of bone formation (G) or resorption (H) markers.

(I) Representative images of double labeling for bone formation on the endosteal surface of the tibia. Green, calcein; red, alizarin red.

(J) Quantification of the mineral apposition rate (MAR) at the endosteal bone surface from double-labeling experiments.

Error bars: SD. *$p < 0.05$, $n = 3$, two-way ANOVA.

Osx$^{+}$ cells derived from MMPs under both basal and teriparatide conditions (Figures 7C and 7D). Here, statistical analysis detected a significant interaction between Igf1r deletion and teriparatide (interaction p value 0.0321), indicating that Igf and teriparatide signaling synergize in promoting osteoblast differentiation. Overall, Igf signaling at least partially mediates the osteogenic function of teriparatide in MMPs.

We next assessed the effect of Igf1r deletion in MMPs on the overall bone anabolic activity of teriparatide after a longer-term treatment. Here we compared CKO or Ctrl littermate mice, both of which received once-daily teriparatide injection for 21 days after TAM administration at one month of age. Analyses of the proximal tibia by μCT detected a significant loss of cancellous bone mass in CKO versus Ctrl mice, regardless of teriparatide (Figures 7E and 7F). Importantly, statistical analysis detected significant interaction between Igf1r and teriparatide (two-way ANOVA, interaction p value 0.0054). Additional quantification revealed that Igf1r deletion reduced Tb.th, coupled with increased Tb.Sp, without altering Tb.N (Figure S7). Serum biochemistry showed that Igf1r deletion suppressed both basal and teriparatide-induced PINP levels, with significant interaction between Igf1r and teriparatide (Figure 7G) (two-way ANOVA, interaction p value 0.0025). However, the deletion did not influence CTX-I levels, which were elevated by teriparatide regardless of Igf1r deletion (Figure 7H). Overall, Igf signaling in MMPs not only contributes to normal trabecular bone formation but also mediates the optimal anabolic response to teriparatide in growing mice.

CellPress
OPEN ACCESS

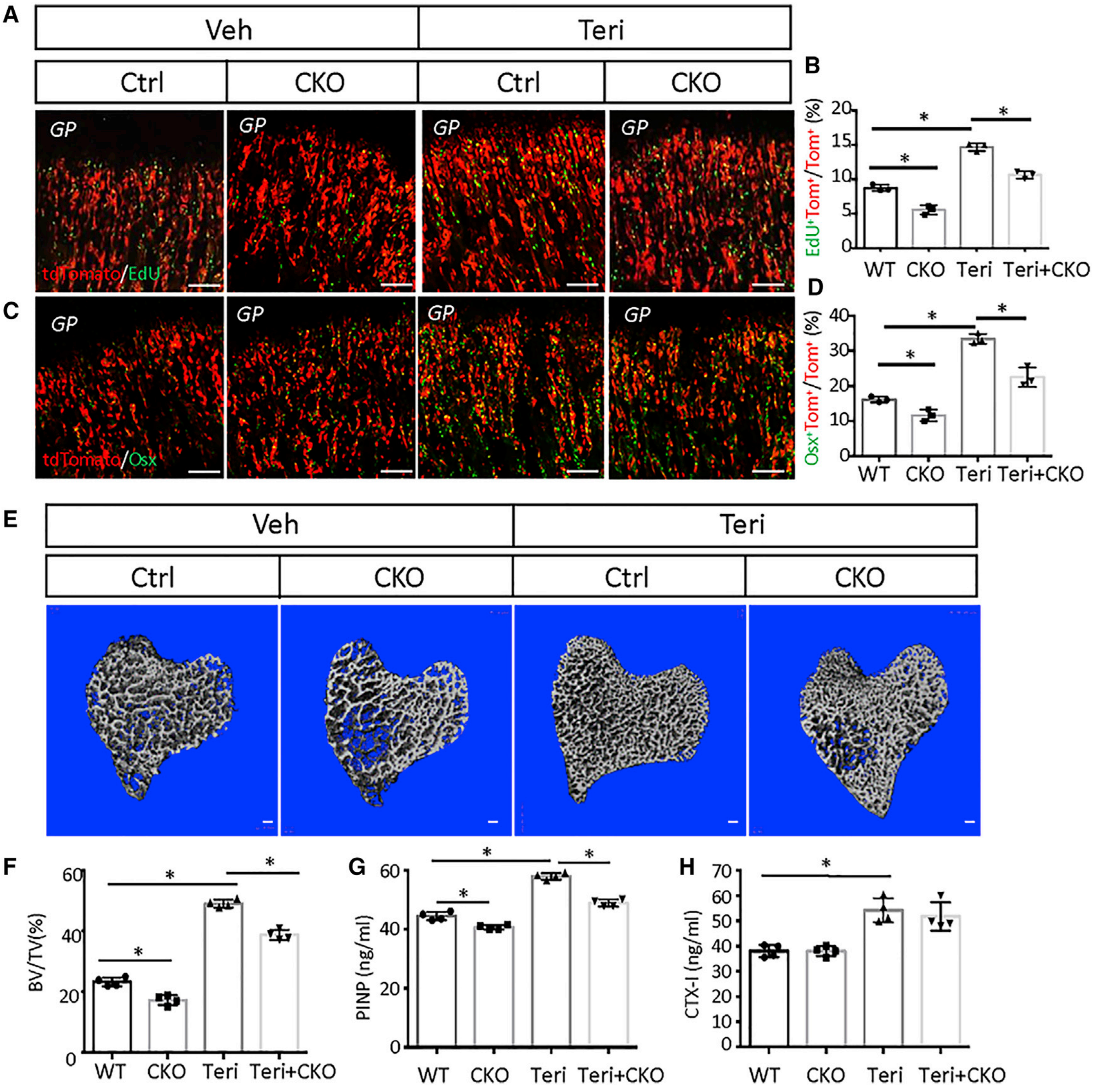


**Figure 7. Igf signaling mediates effects of teriparatide on MMPs and bone formation**

(A and C) Representative confocal images of the chondro-osseous junction in the distal femur. Mice with the genotype of Gli1-CreER$^{T2}$;Ai9;Igf1r$^{c/+}$ (Ctrl) or Gli1-CreER$^{T2}$;Ai9;Igf1r$^{c/c}$ (CKO) were injected with teriparatide or vehicle for three days after TAM treatment. Scale bar: 100 μm.

(B and D) Quantification of tdTomato$^{+}$EdU$^{+}$ (B) or tdTomato$^{+}$Osx$^{+}$ (D) over tdTomato$^{+}$ cells in areas represented by (A) or (C), respectively. Percentage (mean ± SD) was quantified within the primary spongiosa region extending 300 μm from the growth plate of the proximal tibia and spanning the width of the bone, in two sections per mouse and three mice per group. Error bar: SD. *$p < 0.05$, n = 3, two-way ANOVA.

(E) μCT images of cancellous bone of the proximal tibia. Ctrl or CKO mice were injected with teriparatide or vehicle for 21 days. Scale bar: 100 μm.

(F) Quantification of BV/TV by μCT. Error bar: SD. *$p < 0.05$, n = 4, two-way ANOVA.

(G and H) Serum levels of bone formation (G) or resorption (H) markers. Error bar: SD. *$p < 0.05$, n = 4, two-way ANOVA.

## DISCUSSION

The study shows that once-daily teriparatide stimulates both proliferation and osteoblast differentiation of the Gli1$^{+}$ MMPs in postnatal growing mice. The proliferative effect of teriparatide critically depends on Hh, whereas the osteogenic function requires both Hh and Igf signaling in MMPs. scRNA-seq reveals that MMPs are heterogeneous and include a COP that responds to Hh signaling and increases in number when exposed to teriparatide. The study therefore identifies COPs as important target cells of teriparatide within the primary spongiosa of long bones in growing mice.

Besides COP, scRNA-seq has revealed three additional populations (OB, preOB, and MALP) among MMPs or their progenies. Compared with COP, those other populations exhibited little to no expression of the classic Hh target genes, and trajectory analysis predicts that they could derive from Hh-responsive COP. Indeed, lineage tracing with Acan-CreER$^{T2}$ shows that

COP can give rise to OB. However, because Acan-CreER$^{T2}$ marked fewer cells than Gli1-CreER$^{T2}$ in the primary spongiosa at 24 h after TAM induction, COP likely represents only a subset of the MMPs initially marked by Gli1 but maintains Hh responsiveness, whereas the others (e.g., preOB) have lost the response by the time of harvest (6 days after the initial TAM injection). The origin of COP or the other MMPs is uncertain at present but could be the perichondrium, which has been shown to supply trabecular osteoblasts, as well as marrow stromal cells in developing bones (Maes et al., 2010). Alternatively, COP could arise from hypertrophic growth-plate chondrocytes, which have been shown to produce osteoblasts in the primary spongiosa (Yang et al., 2014; Zhou et al., 2014b). However, a recent study shows that the contribution of hypertrophic chondrocytes to trabecular bone mass is minimal after birth (Qin et al., 2020). In contrast, MMPs are a major source for trabecular bone formation in postnatal growing mice (Shi et al., 2017). Therefore, hypertrophic chondrocytes may not be the main source for postnatal MMPs, including COP.

The scRNA-seq data show that three daily injections of teriparatide expand the COP population, apparently at the expense of preOB and MALP without overt changes in OB. Whereas increased proliferation is likely responsible for the COP expansion, the decrease in preOB and MALP may result from changes in differentiation. Because the trajectory analysis indicates that preOB could give rise to either OB or MALP, teriparatide may accelerate the differentiation of preOB in favor of OB over MALP, thus reducing both preOB and MALP populations. Such a model would predict an increase in OBs, but all OBs may not be readily dissociated from the bone matrix, resulting in incomplete representation of OBs in scRNA-seq.

Our finding about COP is consistent with previous reports. The Col2 promoter was previously found to be active in at least a subset of the osteogenic progenitors associated with the trabecular bone during embryonic development (Hilton et al., 2007). Lineage tracing of cells expressing Col2, Sox9, or Acan in postnatal day-3 mice showed that they gave rise to osteoblasts during the subsequent growing phase (Ono et al., 2014). Further studies showed that the Sox9$^{+}$ cells persisted in the metaphysis of long bones in 6-week-old mice and produced osteoblasts with time, although it was not clear whether the Sox9$^{+}$ osteoblast precursors expressed the classic chondrocyte markers (Balani et al., 2017). Here, by scRNA-seq, we detected coexpression of Sox9 with all known chondrocyte markers in the COP cluster, indicating that the Sox9$^{+}$ osteoblast precursors, as previously reported, may overlap significantly with COP. However, despite mRNA expression, the COP cells seem to express little, if any, Sox9 protein detectable by immunostaining. Similarly, others reported only few cells targeted by Sox9-CreER$^{T2}$ in the primary spongiosa to exhibit Sox9 immunostaining (Balani et al., 2017).

The study has revealed important roles of Hh and Igf in mediating the proliferation and differentiation effect of teriparatide on MMPs. We have not explored the molecular mechanism here but have previously shown that Igf signaling augments the Hh signaling output in osteoprogenitors by activating Gli2 through site-specific phosphorylation (Shi et al., 2015). Because teriparatide upregulates Igf1r expression in multiple cell clusters, including COP, it may potentiate Hh signaling in MMPs via Gli2 activation. Although PKA (protein kinase A) activation by teriparatide is expected to dampen Hh signaling, stimulation of Gli2 by Igf signaling could counteract the inhibitory effect and lead to an increase in Hh signaling output (Riobó et al., 2006). Thus, the increased proliferation and osteoblast differentiation of MMPs by teriparatide could be mediated by Igf-induced enhancement of Hh signaling.

The study highlights the pleiotropic effect of teriparatide on skeletal cells. Teriparatide increases trabecular bone formation though stimulation of both proliferation and osteoblast differentiation of MMPs. Interestingly, an early study through *in vivo* competitive binding in the metaphasis of rat long bones identified a cell located in the intertrabecular tissue as a main parathyroid hormone target (Rouleau et al., 1990). The cell, described to be of osteoblast lineage but distinct from a differentiated osteoblast, could be related to MMPs. The expansion of MMPs by teriparatide also helps to explain the previous observation that a single injection of parathyroid hormone increased the number of mesenchymal progenitors that could be isolated from the metaphyseal bone for *in vitro* assays (Siclari et al., 2013). Despite the heterogeneity of MMPs, teriparatide induces in all four populations the expression of osteoblast genes, as well as genes encoding ribosomal proteins and the mitochondrial electron transport chain. The gene expression changes may reflect a general increase in protein synthesis and energy demands in cells responding to teriparatide.

## STAR★METHODS

Detailed methods are provided in the online version of this paper and include the following:

- KEY RESOURCES TABLE
- RESOURCE AVAILABILITY
  - Lead contact
  - Materials availability
  - Data and code availability
- EXPERIMENTAL MODEL AND SUBJECT DETAILS
- METHOD DETAILS
  - Tamoxifen administration
  - Chemicals
  - Immunofluorescence staining
  - Histology and dynamic histomorphometry
  - MicroCT
  - Serum CTX-I and PINP assays
  - Proliferation and apoptosis assay
  - Single-cell RNA sequencing
- QUANTIFICATION AND STATISTICAL ANALYSIS

## SUPPLEMENTAL INFORMATION

Supplemental information can be found online at https://doi.org/10.1016/j.celrep.2021.109542.

## ACKNOWLEDGMENTS

The work is partially supported by NIH grant DK111212 (to F. Long). μCT analysis was conducted at the Penn Center for Musculoskeletal Disorders (PCMD), supported by NIH P30 AR069619.

CellPress
OPEN ACCESS

## AUTHOR CONTRIBUTIONS

Y.S. performed experiments and drafted the paper. X.L., B.Y., F. Lou, and G.H. performed experiments. J.Y.L. and L. Yao analyzed data. J.C., L. Ye, and L.Q. edited the paper. F. Long directed work and wrote the paper.

## DECLARATION OF INTERESTS

The authors declare no competing interests.

Received: February 3, 2021
Revised: May 27, 2021
Accepted: July 26, 2021
Published: August 17, 2021

## REFERENCES

Ahn, S., and Joyner, A.L. (2004). Dynamic changes in the response of cells to positive hedgehog signaling during mouse limb patterning. Cell *118*, 505–516.

Balani, D.H., Ono, N., and Kronenberg, H.M. (2017). Parathyroid hormone regulates fates of murine osteoblast precursors *in vivo*. J. Clin. Invest. *127*, 3327–3338.

Bouxsein, M.L., Boyd, S.K., Christiansen, B.A., Guldberg, R.E., Jepsen, K.J., and Müller, R. (2010). Guidelines for assessment of bone microstructure in rodents using micro-computed tomography. J. Bone Miner. Res. *25*, 1468–1486.

Cao, J., Spielmann, M., Qiu, X., Huang, X., Ibrahim, D.M., Hill, A.J., Zhang, F., Mundlos, S., Christiansen, L., Steemers, F.J., et al. (2019). The single-cell transcriptional landscape of mammalian organogenesis. Nature *566*, 496–502.

Cipriani, C., Irani, D., and Bilezikian, J.P. (2012). Safety of osteoanabolic therapy: a decade of experience. J. Bone Miner. Res. *27*, 2419–2428.

Dietrich, P., Dragatsis, I., Xuan, S., Zeitlin, S., and Efstratiadis, A. (2000). Conditional mutagenesis in mice with heat shock promoter-driven cre transgenes. Mamm. Genome *11*, 196–205.

Esen, E., Lee, S.Y., Wice, B.M., and Long, F. (2015). PTH Promotes Bone Anabolism by Stimulating Aerobic Glycolysis Via IGF Signaling. J. Bone Miner. Res. *30*, 1959–1968.

Henry, S.P., Jang, C.W., Deng, J.M., Zhang, Z., Behringer, R.R., and de Crombrugghe, B. (2009). Generation of aggrecan-CreERT2 knockin mice for inducible Cre activity in adult cartilage. Genesis *47*, 805–814.

Hilton, M.J., Tu, X., and Long, F. (2007). Tamoxifen-inducible gene deletion reveals a distinct cell type associated with trabecular bone, and direct regulation of PTHrP expression and chondrocyte morphology by Ihh in growth region cartilage. Dev. Biol. *308*, 93–105.

Ingham, P.W., and McMahon, A.P. (2001). Hedgehog signaling in animal development: paradigms and principles. Genes Dev. *15*, 3059–3087.

Jilka, R.L. (2007). Molecular and cellular mechanisms of the anabolic effect of intermittent PTH. Bone *40*, 1434–1446.

Joeng, K.S., and Long, F. (2009). The Gli2 transcriptional activator is a crucial effector for Ihh signaling in osteoblast development and cartilage vascularization. Development *136*, 4177–4185.

Kalajzic, I., Kalajzic, Z., Kaliterna, M., Gronowicz, G., Clark, S.H., Lichtler, A.C., and Rowe, D. (2002). Use of type I collagen green fluorescent protein transgenes to identify subpopulations of cells at different stages of the osteoblast lineage. J. Bone Miner. Res. *17*, 15–25.

Kim, S.W., Pajevic, P.D., Selig, M., Barry, K.J., Yang, J.Y., Shin, C.S., Baek, W.Y., Kim, J.E., and Kronenberg, H.M. (2012). Intermittent parathyroid hormone administration converts quiescent lining cells to active osteoblasts. J. Bone Miner. Res. *27*, 2075–2084.

Long, F. (2011). Building strong bones: molecular regulation of the osteoblast lineage. Nat. Rev. Mol. Cell Biol. *13*, 27–38.

Long, F., Zhang, X.M., Karp, S., Yang, Y., and McMahon, A.P. (2001). Genetic manipulation of hedgehog signaling in the endochondral skeleton reveals a direct role in the regulation of chondrocyte proliferation. Development *128*, 5099–5108.

Maes, C., Kobayashi, T., Selig, M.K., Torrekens, S., Roth, S.I., Mackem, S., Carmeliet, G., and Kronenberg, H.M. (2010). Osteoblast precursors, but not mature osteoblasts, move into developing and fractured bones along with invading blood vessels. Dev. Cell *19*, 329–344.

Mizoguchi, T., Pinho, S., Ahmed, J., Kunisaki, Y., Hanoun, M., Mendelson, A., Ono, N., Kronenberg, H.M., and Frenette, P.S. (2014). Osterix marks distinct waves of primitive and definitive stromal progenitors during bone marrow development. Dev. Cell *29*, 340–349.

Mukohira, H., Hara, T., Abe, S., Tani-Ichi, S., Sehara-Fujisawa, A., Nagasawa, T., Tobe, K., and Ikuta, K. (2019). Mesenchymal stromal cells in bone marrow express adiponectin and are efficiently targeted by an adiponectin promoter-driven Cre transgene. Int. Immunol. *31*, 729–742.

Ono, N., Ono, W., Nagasawa, T., and Kronenberg, H.M. (2014). A subset of chondrogenic cells provides early mesenchymal progenitors in growing bones. Nat. Cell Biol. *16*, 1157–1167.

Qin, X., Jiang, Q., Nagano, K., Moriishi, T., Miyazaki, T., Komori, H., Ito, K., Mark, K.V., Sakane, C., Kaneko, H., and Komori, T. (2020). Runx2 is essential for the transdifferentiation of chondrocytes into osteoblasts. PLoS Genet. *16*, e1009169.

Riobó, N.A., Lu, K., Ai, X., Haines, G.M., and Emerson, C.P., Jr. (2006). Phosphoinositide 3-kinase and Akt are essential for Sonic Hedgehog signaling. Proc. Natl. Acad. Sci. USA *103*, 4505–4510.

Rouleau, M.F., Mitchell, J., and Goltzman, D. (1990). Characterization of the major parathyroid hormone target cell in the endosteal metaphysis of rat long bones. J. Bone Miner. Res. *5*, 1043–1053.

Schneider, C.A., Rasband, W.S., and Eliceiri, K.W. (2012). NIH Image to ImageJ: 25 years of image analysis. Nat. Methods *9*, 671–675.

Shi, Y., Chen, J., Karner, C.M., and Long, F. (2015). Hedgehog signaling activates a positive feedback mechanism involving insulin-like growth factors to induce osteoblast differentiation. Proc. Natl. Acad. Sci. USA *112*, 4678–4683.

Shi, Y., He, G., Lee, W.C., McKenzie, J.A., Silva, M.J., and Long, F. (2017). Gli1 identifies osteogenic progenitors for bone formation and fracture repair. Nat. Commun. *8*, 2043.

Siclari, V.A., Zhu, J., Akiyama, K., Liu, F., Zhang, X., Chandra, A., Nah, H.D., Shi, S., and Qin, L. (2013). Mesenchymal progenitors residing close to the bone surface are functionally distinct from those in the central bone marrow. Bone *53*, 575–586.

St-Jacques, B., Hammerschmidt, M., and McMahon, A.P. (1999). Indian hedgehog signaling regulates proliferation and differentiation of chondrocytes and is essential for bone formation. Genes Dev. *13*, 2072–2086.

Yang, L., Tsang, K.Y., Tang, H.C., Chan, D., and Cheah, K.S. (2014). Hypertrophic chondrocytes can become osteoblasts and osteocytes in endochondral bone formation. Proc. Natl. Acad. Sci. USA *111*, 12097–12102.

Zhong, L., Yao, L., Tower, R.J., Wei, Y., Miao, Z., Park, J., Shrestha, R., Wang, L., Yu, W., Holdreith, N., et al. (2020). Single cell transcriptomics identifies a unique adipose lineage cell population that regulates bone marrow environment. eLife *9*, e54695.

Zhou, B.O., Yue, R., Murphy, M.M., Peyer, J.G., and Morrison, S.J. (2014a). Leptin-receptor-expressing mesenchymal stromal cells represent the main source of bone formed by adult bone marrow. Cell Stem Cell *15*, 154–168.

Zhou, X., von der Mark, K., Henry, S., Norton, W., Adams, H., and de Crombrugghe, B. (2014b). Chondrocytes transdifferentiate into osteoblasts in endochondral bone during development, postnatal growth and fracture healing in mice. PLoS Genet. *10*, e1004820.

# STAR★METHODS

## KEY RESOURCES TABLE

| REAGENT or RESOURCE | SOURCE | IDENTIFIER |
|---|---|---|
| Antibodies | | |
| Sp7/Osterix | Abcam | Ab22552; RRID:AB_2194492 |
| Runx2 (D1L7F) Rabbit mAb | Cell Signaling Technology | 12556; RRID:AB_2732805 |
| Gli1 | Novus Biologicals | Nb600-600; RRID:AB_2111758 |
| Sox9 | Millipore Sigma | AB5535; RRID: AB_2239761 |
| Goat anti-rabbit Alexa Fluor 488 | Thermo-Fisher Scientific | A27034; RRID:AB_2536097 |
| Goat anti-rabbit Alexa Fluor 647 | Thermo-Fisher Scientific | A27040; RRID:AB_2536101 |
| Chemicals, peptides, and recombinant proteins | | |
| Tamoxifen | Sigma | T5648 |
| PTH (1-34) (human) Acetate | Bachem Biosciences | H-4835-GMP, 4033364 |
| Tissue-Tek® O.C.T. Compound | Vavantor | 4583 |
| GDC-0449 | LC laboratories | V-4050 |
| DPBS | Thermo | 14190250 |
| FBS | Thermo | 26140087 |
| DAPI | Vector Laboratories | H-1200-10 |
| Critical commercial assays | | |
| RatLaps ELISA | Immunodiagnostic Systems | AC-02F1 |
| Rat/Mouse PINP EIA Kit | Immunodiagnostic Systems | AC-33F1 |
| Click-iT EdU Alexa Fluor 488 Imaging Kit | Invitrogen | C10337 |
| DeadEndTM fluorometric TUNEL System | Promega | G3250 |
| Deposited data | | |
| Raw and analyzed data | This paper | GEO: GSE169560 |
| Experimental models: Organisms/strains | | |
| Mouse: Gli1tm3(cre/ERT2)Alj/J (common name: Gli1-CreER$^{T2}$) | The Jackson Laboratory | 007913 |
| Mouse: B6.Cg-Acantm1(cre/ERT2)Crm/J (common name: Acan-CreER$^{T2}$) | The Jackson Laboratory | 019148 |
| Mouse: B6.Cg-Gt(ROSA)26Sortm9(CAG-tdTomato)Hze/J (common name: Ai9) | The Jackson Laboratory | 007909 |
| Mouse: B6.Cg-Tg(Col1a1*2.3-GFP)1Rowe/J (common name: ColI-GFP) | The Jackson Laboratory | 013134 |
| Mouse: 129-Ihhtm1Amc/J (common name: Ihh$^{+/-}$) | The Jackson Laboratory | 004290 |
| Mouse: B6;129-Igf1rtm2Arge/J (common name: Igf1r$^{c/c}$) | The Jackson Laboratory | 012251 |
| Software and algorithms | | |
| BIOQUANT Image Analysis System | BIOQUANT Image Analysis Corporation | BIOQUANT Osteo II |
| ImageJ | (Schneider et al., 2012) | https://imagej.nih.gov/ij/ |

## RESOURCE AVAILABILITY

### Lead contact

Further information and requests for resources and reagents should be directed to and will be fulfilled by the Lead Contact, Fanxin Long (longf1@chop.edu).

### Materials availability

This study did not generate new unique reagents.

### Data and code availability

- The single-cell RNA-seq data generated during this study are available at Gene Expression Omnibus (GEO: GSE169560, https://www.ncbi.nlm.nih.gov/geo/query/acc.cgi?acc=GSE169560)
- This paper does not report original code.
- Any additional information required to reanalyze the data reported in this paper is available from the lead contact upon request.

## EXPERIMENTAL MODEL AND SUBJECT DETAILS

Mouse strains Gli1-CreER$^{T2}$, Acan-CreER$^{T2}$, Ai9, CollI-GFP, Ihh$^{+/-}$ and Igf1r$^{c/c}$ are as described (Ahn and Joyner, 2004; Dietrich et al., 2000; Henry et al., 2009; Kalajzic et al., 2002; St-Jacques et al., 1999). Acan-CreER$^{T2}$ and Ai9 are in the C57BL6 genetic background. Gli1-CreER$^{T2}$, Igf1r$^{c/c}$ and Ihh$^{-/-}$ are mostly C57BL6 mixed with 129. ColI-GFP is mostly C57BL6 mixed with CD-1. All mice were maintained in a specific pathogen-free facility with a 12-hr light cycle (6 am −6 pm) and standard chow diet (Lab Diet, 5015). Both male and female mice were used with no sex difference observed unless otherwise indicated. Mice were injected with TAM at four weeks (for Gli1-CreER$^{T2}$ studies) or one month (for Acan-CreER$^{T2}$ studies) of age and then harvested at indicated times. All mouse studies were approved by the Institutional Animal Care and Use Committee (IACUC) at The Children's Hospital of Philadelphia.

## METHOD DETAILS

### Tamoxifen administration

Tamoxifen (TAM) (Sigma) dissolved in corn oil was administered by oral gavage once daily for three consecutive days unless otherwise indicated. TAM dose of 4 mg/30 g body weight was used except for Acan-CreER$^{T2}$ experiments where 3 mg/30 g was used. Both males and females were used in lineage tracing experiments and no sex-dependent difference was observed. Sex-matched littermates were used for comparison between experimental groups.

### Chemicals

PTH (1-34) (human) Acetate (H-4835-GMP, 4033364), also known as teriparatide, was purchased from Bachem Biosciences (Torrance, CA, USA). Teriparatide was dissolved in a sterile aqueous solution containing 0.9% NaCl, 0.1% BSA, 0.001 N HCl. This solution was used as vehicle control. Teriparatide was injected intraperitoneally once daily at 400 ng per gram of body weight. GDC-0449 (Vismodegib, LC laboratories, V-4050) was injected intraperitoneally twice a day with a 6-hr interval at 1.5 mg per 30 g of body weight for indicated days.

### Immunofluorescence staining

Dissected bones were fixed with 4% paraformaldehyde (PFA) overnight at room temperature and partially decalcified in 14% EDTA for 3 days with daily changes of solution. The bones were then infiltrated with 30% sucrose overnight at 4°C for cryoprotection and embedded in optimal cutting temperature (Tissue-Tek). Sections of 10-μm thickness were prepared with a Leica cryostat equipped with Cryojane (Leica, IL). The sections were stained with the primary antibody for Osx (ab22552, Abcam, 1:200), Runx2 (12556, CST, 1:500), Gli1 (NB600-600, Novus, 1:50), or Sox9 (#AB5535, Millipore Sigma, 1:200) and then the secondary antibody goat anti-rabbit Alexa Fluor 488 or goat anti-rabbit Alexa Fluor 647 (Thermo- Fisher Scientific, 1:500). Slides were mounted with anti-fade mounting medium with DAPI (Vector Laboratories), and images were acquired with a Leica confocal microscope.

### Histology and dynamic histomorphometry

For histology, tibiae were isolated from mice and fixed in 10% buffered formalin overnight at room temperature, followed by decalcification in 14% EDTA for 2 weeks. After decalcification, tibiae were processed for paraffin embedding and then sectioned at 6-μm thickness. Hematoxylin and eosin (H&E) staining was performed on paraffin sections. For dynamic histomorphometry, calcein green or alizarin red (10 mg/kg; Sigma, Saint Louis, MO, USA) were injected intraperitoneally on 7 days and 2 days before sacrificing the mice and femur were harvested. Bone were fixed with 4% PFA for 24hrs and then infiltrated with 30% sucrose overnight at 4°C for cryoprotection and embedded in optimal cutting temperature (Tissue-Tek). Sections of 10-μm thickness were prepared with a Leica cryostat (Leica, IL) and Cryoflim type II membrane (Section Lab, Co. Ltd., Japan). Double labeling parameters were acquired with a BIOQUANT Osteo II from 3 sections per mouse and 3 mice for each genotype. The specific bone surfaces being quantified are indicated in figure legends.

### MicroCT

The tibias were scanned with μCT 35 (Scanco Medical AG) according to recommendations of American Society of Bone and Mineral Research (Bouxsein et al., 2010). For quantification of trabecular bone parameters, 100 CT slices (1.6 mm total) immediately below the proximal tibial or distal femoral growth plate were analyzed.

### Serum CTX-I and PINP assays

Serum was collected from mice after 6 hours of fasting for CTX-I and PINP assays. The assays were performed with the RatLaps ELISA or Rat/Mouse PINP EIA Kit (both from Immunodiagnostic Systems, Ltd.).

### Proliferation and apoptosis assay

EdU was injected intraperitoneally at 10 μg/g body weight at 4 h before harvest. Frozen sections of bones were prepared as above; EdU signals were detected with the Click-iT EdU Alexa Fluor 488/647 Imaging Kit (Invitrogen). Apoptotic cells were detected by TUNEL assay with DeadEnd$^{TM}$ fluorometric TUNEL System (G3250, Promega).

### Single-cell RNA sequencing

Gli1-CreER$^{T2}$; Ai9 mice at one month of age were administrated TAM for three consecutive days once daily followed by three daily injections of teriparatide or vehicle (n = 5 per group, 3 males and 2 females). Cells from the cancellous bone region of the femur were isolated as follows. Upon removal of the epiphysis, the metaphyseal bone region extending about 5 mm below the growth plate was dissected and crushed with mortar and pestle in FACS Buffer, followed by digestion with 0.25% collagenase type I (C0130, Sigma-Aldrich) in PBS (GIBCO) for 20 min at 37°C with gentle shaking. The cells were then centrifuged and re-suspended with FACS Buffer and filtered with a 70-μm cell strainer. The tdTomato$^{+}$CD31$^{-}$CD45$^{-}$Ter119$^{-}$ cells were sorted with Navios EX (Beckman Coulter). Cell viability and number were determined with a cell counter (Biorad).

10x Genomics Chromium platform was used to capture and barcode cells to generate single-cell Gel Beads-in-Emulsion (GEMs) by following the manufacturer's protocol. The oligonucleotides coating the gel beds enable mRNA capture by 30 bp oligo-dT and provide barcodes to index cells (16 bp) as well as transcripts (10 bp unique molecular identifier; UMI). Following reverse transcription, cDNAs with both barcodes were amplified, and a library was constructed using the Single Cell 3′ Reagent Kit (v2 chemistry) for each sample. The resulting libraries were sequenced on an Illumina NovaSeq 6000 System. Sample demultiplexing, barcode processing and UMI counting were performed by using the official 10x Genomics pipeline Cell Ranger v2.1.0 (https://support.10xgenomics.com). Briefly, FASTQs generated from Illumina sequencing output were aligned to the mouse reference genome (mm10). Next, Gene-Barcode matrices were generated for each individual sample by counting unique molecular identifiers (UMIs) and filtering non-cell associated barcodes. Finally, we generated a gene-barcode matrix containing the barcoded cells and gene expression counts. The resulting gene-cell UMI count matrices for each sample were then concatenated into one matrix using the "cellranger aggr" pipeline, which also normalized the libraries to the same sequencing depth. All downstream single-cell analyses were performed using Cell Ranger and Seurat by default setting. The filtered gene-barcode matrix of each mouse identified by Cell Ranger Count was inputted into Seurat. Doublets or cells with poor quality (genes > 6000, genes < 200, or > 10% genes mapping to mitochondrial genome) were excluded. To avoid batch differences, the Seurat alignment method canonical correlation analysis (CCA) was used for integrated analysis of datasets. For clustering, highly variable genes were selected and the principal components based on those genes used to build a graph, which was segmented with a resolution of 0.6. Based on filtered gene expression matrix by Seurat, between samples differential expression analysis was carried out using the edgeR package to obtain zone-specific marker genes. Genes with a p value < 0.05 and absolute fold change ≥ 1.5 were regarded as differentially expressed genes. The trajectory and pseudotime analysis was performed with the algorithms of Monocle package V3 (Cao et al., 2019). The sequencing data is deposited in NCBI GEO: GSE169560.

## QUANTIFICATION AND STATISTICAL ANALYSIS

Statistical significance is calculated with Student's t test or two-way ANOVA as indicated in figure legends. P value of > 0.05 is considered statistically significant. N refers to number of mice quantified. Error bars denote standard deviation.

Cell Reports, Volume *36*

## Supplemental information

# Gli1$^{+}$ progenitors mediate bone anabolic function of teriparatide via Hh and Igf signaling

**Yu Shi, Xueyang Liao, James Y. Long, Lutian Yao, Jianquan Chen, Bei Yin, Feng Lou, Guangxu He, Ling Ye, Ling Qin, and Fanxin Long**

**Supplemental Information**

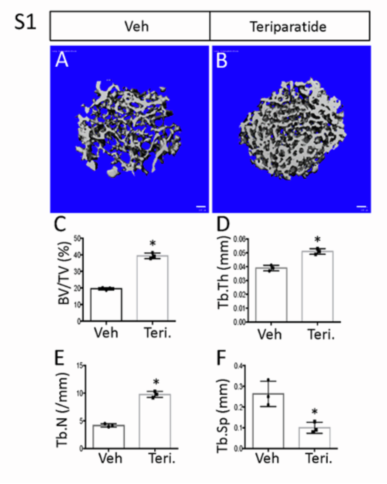


**Figure S1. Related to Fig. 1. Teriparatide increases trabecular bone mass in growing mice.**
One-month-old mice with the genotype of Gli1-CreER$^{T2}$; Ai9; Col1GFP were injected daily with teriparatide for 21 days after TAM induction.
(A, B) 3-D reconstruction of trabecular bone of the distal femur by microCT. Scale bars, 100 µm.
(C-F) Trabecular bone parameters of the distal femur by microCT. BV/TV, bone volume over total volume; Tb.Th, trabecular thickness; Tb.N, trabecular number; Tb.Sp, trabecular separation. *: p<0.05, n=3, student's t-test. Error bars: SD.

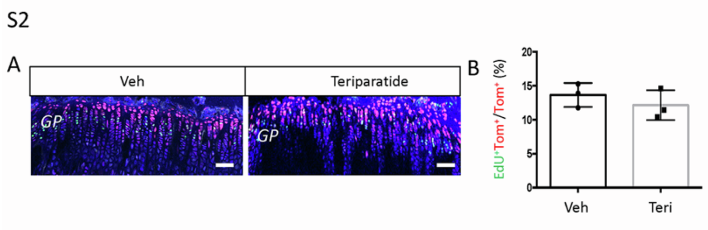


**Figure S2. Related to Fig. 1. Teriparatide does not affect chondrocyte proliferation.**
(A) Representative confocal images of the growth plate in proximal tibia of Gli1CreER$^{T2}$;Ai9 mice injected with teriparatide or veh for 3 days after TM. Scale bar, 100 µm.
(B) Quantification of EdU labeling among tdTomato$^{+}$ cells. N=3. Error bar: std.

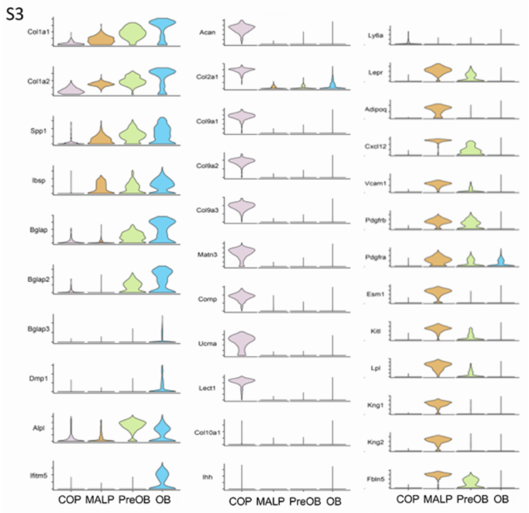

**Figure S3. Related to Fig. 2. Violin plots of selected gene expression in various t-SNE clusters.**

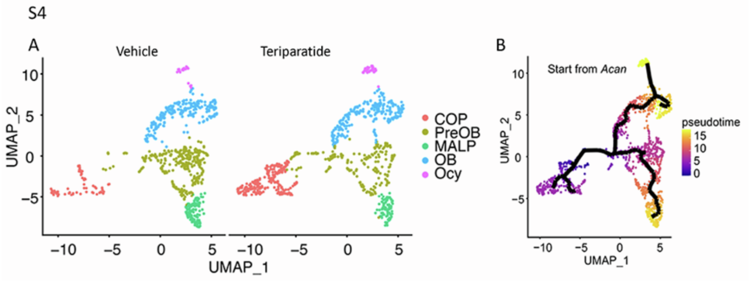


**Figure S4. Related to Fig. 2. Clustering and trajectory analysis of scRNA-seq data.**
(A) UMAP clustering showing expansion of COP population by teriparatide. (B) Trajectory analysis of integrated data based on with Monocle 3.

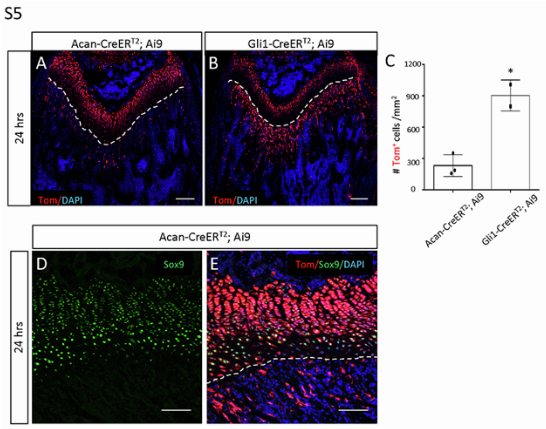


**Figure S5. Related to Fig. 3. Acan-CreER$^{T2}$ targets COP in primary spongiosa.**
(A, B) Representative images of longitudinal sections through the distal end of femur from mice with indicated genotype. The mice were dosed with TAM once daily for three days starting at 28 days of age and harvested 24 hrs after last dosing. Dotted line denotes the boundary between growth plate and primary spongiosa. Scale bar: 200 µm.
(C) Quantification of tdTomato+ cells within the primary spongiosa region (200 µm below the growth plate and across the width from periosteum on one side to the other). *: $p<0.05$, student's t test, N= 2 or 3 as indicated. Error bars: SD.
(D, E) Immunofluorescence staining of Sox9 on longitudinal sections of distal femur from mice dosed in the same way as above. Dotted line denotes boundary between growth plate and primary spongiosa. Scale bar: 100 µm.

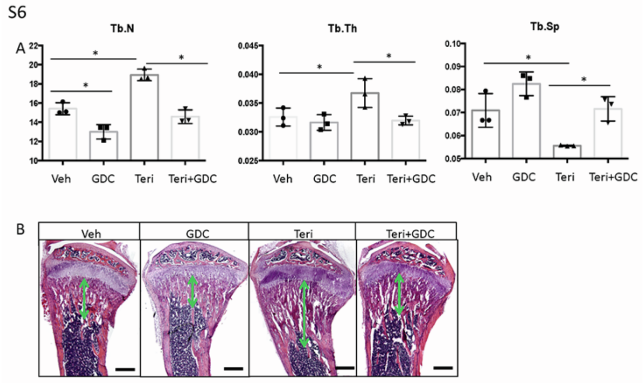


**Figure S6. Related to Fig. 6. Hh inhibitor abolishes bone anabolic effect of teriparatide.**
(A) Trabecular bone parameters of proximal tibia by µCT in wild type mice with indicated treatments for 10 days.
*: $p<0.05$, n=3, two-way ANOVA. Error bars: SD.

(B) H&E staining of longitudinal sections through the proximal tibia. Double-headed arrows denote trabecular bone region. Scale bar: 500 µm.

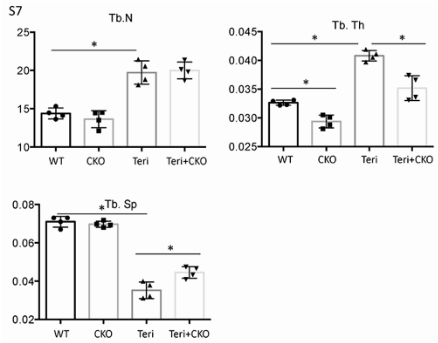


**Figure S7. Related to Fig. 7. Igf signaling in MMPs mediates bone anabolic effect of teriparatide.** Trabecular bone parameters in proximal tibia by µCT. Tb.Th, trabecular thickness; Tb.N, trabecular number; Tb. Sp, trabecular separation. *: $p<0.05$, $n=4$, two-way ANOVA. Error bars: SD.

**Table S1. Osteoblast genes upregulated by teriparatide (adj. p value <0.05), related to figure 2**

| | | OB | | |
|---|---|---|---|---|
| **Gene_name** | **Teriparatide** | **Ctrl** | **log2FoldChange** | **p value adj.** |
| **Mepe** | 9.0777407 | 1.5984315 | 2.40864179 | 3.27E-06 |
| **Dmp1** | 17.5897159 | 6.64350728 | 1.384023316 | 0.00072508 |
| **Spp1** | 318.871648 | 132.344947 | 1.267840155 | 0.00023216 |
| **Sost** | 0.18145602 | 4.16E-17 | 1.232158166 | 2.03E-06 |
| **Ibsp** | 35.1029097 | 17.2279159 | 1.023080885 | 7.48E-05 |
| **Phex** | 2.97786455 | 1.53991044 | 0.903718112 | 0.00580263 |
| **Igf1r** | 0.51172502 | 0.26877033 | 0.712284935 | 0.00061673 |
| **Runx3** | 0.24985181 | 0.11985354 | 0.685275545 | 0.01811181 |
| **Runx2** | 1.63327506 | 1.10661507 | 0.517875749 | 0.0049993 |
| | | **COP** | | |
| **Gene_name** | **Teriparatide** | **Ctrl** | **log2FoldChange** | **p value adj.** |
| **Spp1** | 79.6257212 | 5.6656089 | 3.765964542 | 3.57E-05 |
| **Bglap** | 7.12849537 | 1.5878098 | 2.064941224 | 0.00122549 |
| **Bglap2** | 3.61905627 | 0.9253039 | 1.805996461 | 0.01239873 |
| **Pth1r** | 21.857693 | 11.7368716 | 0.889462793 | 0.00122549 |
| **Mgp** | 1535.63859 | 830.105372 | 0.887362707 | 0.0015243 |
| **Runx3** | 8.44393483 | 5.02217675 | 0.727641422 | 0.00028417 |
| **Igf1r** | 3.55561895 | 2.44633204 | 0.501523514 | 0.00877632 |
| | | **MALP** | | |
| **Gene_name** | **Teriparatide** | **Ctrl** | **log2FoldChange** | **p value adj.** |
| **Igf1r** | 1.41764315 | 0.58445898 | 1.12093741 | 1.83E-06 |
| **Runx3** | 0.4429948 | 0.20982488 | 0.755811899 | 0.03278384 |
| **Runx2** | 3.38584968 | 2.06855003 | 0.66985307 | 0.00018058 |
| **Col1a2** | 32.5810338 | 21.5872848 | 0.591683594 | 0.0001039 |
| **Runx1** | 9.41439148 | 7.29164917 | 0.356351682 | 0.04347791 |
| | | **PreOB** | | |
| **Gene_name** | **Teriparatide** | **Ctrl** | **log2FoldChange** | **p value adj.** |
| **Ibsp** | 35.1973347 | 2.87881181 | 3.553566065 | 5.14E-18 |
| **Spp1** | 158.306014 | 15.2091273 | 3.370175069 | 6.12E-22 |
| **Mgp** | 7.29253118 | 1.42200914 | 2.265824032 | 2.74E-10 |
| **Mmp13** | 28.2094678 | 5.82654048 | 2.245066959 | 2.72E-09 |
| **Enpp1** | 0.60317565 | 0.02809828 | 2.222976672 | 1.06E-08 |
| **Dmp1** | 0.52975239 | 1.39E-17 | 2.191031879 | 1.14E-05 |
| **Postn** | 8.55677376 | 2.27527289 | 1.859864679 | 4.84E-06 |
| **Col1a2** | 116.207557 | 41.9731649 | 1.466451914 | 4.43E-08 |
| **Sparc** | 61.3568624 | 24.71528 | 1.307552181 | 7.71E-07 |
| **Col1a1** | 136.120292 | 57.6667035 | 1.23744936 | 0.00079425 |
| **Bgn** | 13.8732873 | 6.28057894 | 1.125676081 | 1.56E-09 |
| **Runx1** | 2.69878894 | 1.4830065 | 0.815731883 | 0.00199852 |

**Table S2. Gene Ontology Enrichment by Teriparatide in COP, Related to Figure 2**

| Annotation Cluster 1 | Enrichment Score: 40.72 | Count | P_Value | Fold Change | Benjamini |
|---|---|---|---|---|---|
| UP_KEYWORDS | Ribonucleoprotein | 66 | 1.70E-49 | 1.20E+01 | 4.90E-47 |
| KEGG_PATHWAY | Ribosome | 54 | 1.10E-47 | 1.40E+01 | 2.10E-45 |
| UP_KEYWORDS | Ribosomal protein | 55 | 6.30E-47 | 1.50E+01 | 9.00E-45 |
| GOTERM_MF_DIRECT | Structural constituent of ribosome | 56 | 3.10E-39 | 1.00E+01 | 1.60E-36 |
| GOTERM_CC_DIRECT | Ribosome | 49 | 6.50E-39 | 1.30E+01 | 2.10E-36 |
| GOTERM_BP_DIRECT | Translation | 53 | 2.00E-26 | 6.30E+00 | 3.60E-23 |
| **Annotation Cluster 2** | **Enrichment Score: 18.24** | **Count** | **P_Value** | **Fold Change** | **Benjamini** |
| KEGG_PATHWAY | Oxidative phosphorylation | 37 | 3.20E-26 | 9.80E+00 | 3.00E-24 |
| KEGG_PATHWAY | Parkinson's disease | 34 | 9.90E-22 | 8.40E+00 | 6.20E-20 |
| UP_KEYWORDS | Mitochondrion inner membrane | 33 | 1.10E-17 | 7.00E+00 | 5.40E-16 |
| KEGG_PATHWAY | Non-alcoholic fatty liver disease | 30 | 6.60E-17 | 7.10E+00 | 3.10E-15 |
| KEGG_PATHWAY | Huntington's diease | 32 | 7.90E-16 | 6.00E+00 | 3.00E-14 |
| KEGG_PATHWAY | Alzheimer's disease | 30 | 2.10E-15 | 6.30E+00 | 6.40E-14 |
| **Annotation Cluster 3** | **Enrichment Score: 16.89** | **Count** | **P_Value** | **Fold Change** | **Benjamini** |
| UP_KEYWORDS | Respiratory chain | 21 | 6.50E-20 | 1.80E+01 | 4.70E-18 |
| GOTERM_CC_DIRECT | Respiratory chain | 19 | 5.70E-17 | 1.60E+01 | 3.10E-15 |
| UP_KEYWORDS | Electron transport | 22 | 5.70E-16 | 1.10E+01 | 2.30E-14 |

**Table S3. Gene Ontology Enrichment by Teriparatide in MALP, Related to Figure 2**

| Annotation Cluster 1 | Enrichment Score: 45.39 | Count | P_Value | Fold Change | Benjamini |
|---|---|---|---|---|---|
| KEGG_PATHWAY | Ribosome | 68 | 4.40E-58 | 1.20E+01 | 9.60E-56 |
| UP_KEYWORDS | Ribosomal protein | 69 | 3.40E-55 | 1.20E+01 | 1.00E-52 |
| GOTERM_CC_DIRECT | Ribosome | 65 | 2.10E-49 | 1.10E+01 | 9.20E-47 |
| UP_KEYWORDS | Ribonucleoprotein | 74 | 2.40E-47 | 8.80E+00 | 3.70E-45 |
| GOTERM_MF_DIRECT | structural constituent of ribosome | 71 | 2.40E-45 | 8.60E+00 | 1.60E-42 |
| GOTERM_CC_DIRECT | intracellular ribonucleoprotein complex | 65 | 7.20E-34 | 6.60E+00 | 1.50E-31 |
| GOTERM_BP_DIRECT | translation | 72 | 1.40E-33 | 5.70E+00 | 3.60E-30 |
| **Annotation Cluster 2** | **Enrichment Score: 13.37** | **Count** | **P_Value** | **Fold Change** | **Benjamini** |
| KEGG_PATHWAY | Oxidative phosphorylation | 36 | 6.20E-20 | 6.80E+00 | 6.80E-18 |
| KEGG_PATHWAY | Parkinson's disease | 32 | 3.40E-15 | 5.60E+00 | 2.50E-13 |
| KEGG_PATHWAY | Alzheimer's disease | 32 | 4.90E-13 | 4.70E+00 | 2.70E-11 |
| KEGG_PATHWAY | NAFLD | 28 | 2.60E-11 | 4.70E+00 | 1.20E-09 |
| KEGG_PATHWAY | Huntington's disease | 31 | 5.40E-11 | 4.10E+00 | 2.00E-09 |
| **Annotation Cluster 3** | **Enrichment Score: 12.65** | **Count** | **P_Value** | **Fold Change** | **Benjamini** |
| UP_KEYWORDS | Respitatory chain | 20 | 2.00E-15 | 1.20E+01 | 1.20E-13 |
| GOTERM_CC_DIRECT | respiratory chain | 19 | 6.40E-14 | 1.10E+01 | 3.10E-12 |
| UP_KEYWORDS | Electron transport | 20 | 8.30E-11 | 6.80E+00 | 3.60E-09 |

## Table S4. Gene Ontology Enrichment by Teriparatide in PreOB, Related to Figure 2

| Annotation Cluster 1 | Enrichment Score: 24.87 | Count | P_Value | Fold Change | Benjamini |
|---|---|---|---|---|---|
| UP_KEYWORDS | Ribonucleoprotein | 55 | 3.70E-28 | 6.50E+00 | 6.50E-26 |
| UP_KEYWORDS | Ribosomal protein | 46 | 4.00E-28 | 8.30E+00 | 6.50E-26 |
| KEGG_PATHWAY | Ribosome | 45 | 1.30E-26 | 7.30E+00 | 3.10E-24 |
| GOTERM_CC_DIRECT | ribosome | 42 | 2.00E-23 | 7.20E+00 | 3.40E-21 |
| GOTERM_MF_DIRECT | structural constituent of ribosome | 47 | 1.10E-21 | 5.70E+00 | 8.00E-19 |
| Annotation Cluster 2 | Enrichment Score: 8.41 | Count | P_Value | Fold Change | Benjamini |
| INTERPRO | Collagen triple helix repeat | 18 | 5.00E-11 | 8.10E+00 | 5.80E-08 |
| UP_KEYWORDS | Collagen | 18 | 1.20E-10 | 7.80E+00 | 3.80E-09 |
| GOTERM_CC_DIRECT | collagen trimer | 18 | 5.30E-10 | 7.00E+00 | 1.90E-08 |
| KEGG_PATHWAY | Protein digestion and absorption | 14 | 7.40E-05 | 3.80E+00 | 1.40E-03 |
| Annotation Cluster 3 | Enrichment Score: 7.82 | Count | P_Value | Fold Change | Benjamini |
| KEGG_PATHWAY | Oxidative phosphorylation | 27 | 9.70E-11 | 4.60E+00 | 1.10E-08 |
| KEGG_PATHWAY | Alzheimer's disease | 28 | 4.80E-09 | 3.70E+00 | 2.80E-07 |
| KEGG_PATHWAY | Parkinson's disease | 25 | 1.20E-08 | 4.00E+00 | 5.60E-07 |
| KEGG_PATHWAY | Huntington's disease | 24 | 9.20E-06 | 2.90E+00 | 2.40E-04 |

## Table S5. Gene Ontology Enrichment by Teriparatide in OB, Related to Figure 2

| Annotation Cluster 1 | Enrichment Score: 30.53 | Count | P_Value | Fold Change | Benjamini |
|---|---|---|---|---|---|
| UP_KEYWORDS | Ribonucleoprotein | 57 | 4.70E-36 | 8.80E+00 | 1.40E-33 |
| UP_KEYWORDS | Ribosomal protein | 48 | 2.10E-35 | 1.10E+01 | 3.10E-33 |
| KEGG_PATHWAY | Ribosome | 47 | 3.50E-34 | 1.00E+01 | 6.90E-32 |
| GOTERM_MF_DIRECT | Structural constituent of ribosome | 51 | 6.60E-31 | 8.20E+00 | 3.90E-28 |
| GOTERM_CC_DIRECT | Ribosome | 44 | 2.70E-30 | 1.00E+01 | 1.00E-27 |
| GOTERM_BP_DIRECT | Translation | 49 | 1.00E-20 | 5.20E+00 | 2.20E-17 |
| Annotation Cluster 2 | Enrichment Score: 8.61 | Count | P_Value | Fold Change | Benjamini |
| UP_KEYWORDS | Respiratory chain | 14 | 4.10E-10 | 1.10E+01 | 2.10E-08 |
| GOTERM_CC_DIRECT | Respiratory chain | 14 | 6.60E-10 | 1.00E+01 | 2.50E-08 |
| UP_KEYWORDS | Electron transport | 15 | 5.30E-08 | 6.70E+00 | 1.80E-06 |
| Annotation Cluster 3 | Enrichment Score: 2.61 | Count | P_Value | Fold Change | Benjamini |
| INTERPRO | Ribonucleoprotein LSM domain | 5 | 9.00E-04 | 1.10E+01 | 2.10E-01 |
| SMART | Sm | 5 | 1.10E-03 | 1.10E+01 | 2.30E-01 |
| INTERPRO | Like-Sm (LSM) domain | 5 | 1.60E-03 | 9.70E+00 | 2.10E-01 |
| GOTERM_CC_DIRECT | U12-type spliceosomal complex | 4 | 2.50E-02 | 6.30E+00 | 2.50E-01 |
